# Multi-omics resolved integration reveals microbial niche separation in soil aggregates

**DOI:** 10.1093/ismeco/ycag161

**Published:** 2026-06-11

**Authors:** Jonathan Y Lin, Júlia Brandão Gontijo, Cameron K McMillan, Jane D Fudyma, Daoyuan Wang, Erika H Yao, Jordan M Sayre, Joanne B Emerson, David A Lipson, Cristina Lazcano, Kate M Scow, Jorge L Mazza Rodrigues

**Affiliations:** Department of Land, Air and Water Resources, University of California, Davis, CA 95616, United States; Department of Land, Air and Water Resources, University of California, Davis, CA 95616, United States; Department of Land, Air and Water Resources, University of California, Davis, CA 95616, United States; Department of Plant Pathology, University of California, Davis, CA 95616, United States; Department of Environmental Science and Engineering, Shanghai University, Shanghai 200444, People’s Republic of China; Department of Land, Air and Water Resources, University of California, Davis, CA 95616, United States; Department of Land, Air and Water Resources, University of California, Davis, CA 95616, United States; Department of Plant Pathology, University of California, Davis, CA 95616, United States; Department of Biology, San Diego State University, San Diego, CA 92182, United States; Department of Land, Air and Water Resources, University of California, Davis, CA 95616, United States; Department of Land, Air and Water Resources, University of California, Davis, CA 95616, United States; Department of Land, Air and Water Resources, University of California, Davis, CA 95616, United States; Environmental Genomics and Systems Biology Division, Lawrence Berkeley National Laboratory, Berkeley, CA 94720, United States

**Keywords:** soil aggregates, metagenome, ammonia-oxidizing archaea, microenvironment, metabolomics, MAGs

## Abstract

The soil matrix is a heterogeneous mixture composed of aggregates—three-dimensional complexes composed of organic materials and mineral particles. Soil aggregates vary considerably in physical and chemical properties by size, making them unique habitats for distinct microbial communities and metabolic pathways. Yet, this microscale spatial variability is often overlooked in studies that use homogenized soil cores. We investigated the microbial taxonomy, functional gene composition, and metabolic products observed in four aggregate size fractions ranging from 8 mm to free particles (below 53 μm) collected from agricultural soils under two different management practices. The functional gene composition differed significantly among aggregate sizes, with higher abundances of genes for the degradation of plant-derived compounds in the macroaggregates and for biomass recycling in the two smallest size fractions. These differences were corroborated by significant differences in the composition of the metabolome but not in specific enzyme activities. Both taxonomic profiling and reconstruction of genomes from metagenomes revealed a higher abundance of ammonia-oxidizing archaea in the macroaggregates in comparison to other aggregate sizes, and analysis of their genomes revealed complementary metabolisms potentially enabling them to colonize different niches within the same habitat. Together, our results show that soil microbial communities and their functions are shaped by the size of soil aggregates, likely driven by differences in resource availability between macro- and microaggregates.

## Introduction

Soils harbor complex assemblages of resident bacteria, archaea, and eukaryotes that together play fundamental roles in sustaining global biogeochemical cycles [1] and supporting plant [2] and human health [3]. In the face of climate change and a growing population, the soil microbiome and its functions represent a critical biotic tool that can be leveraged to meet these challenges [4, 5]. However, despite the use of modern high-throughput sequencing techniques and numerous studies characterizing soil microbiomes across land uses [6, 7], rising temperatures [8–10], and moisture regimes [11, 12], a consistent foundational understanding of how specific community compositions relate to functional potential remains elusive. Soil microbiome composition does not always predict specific functions [1], and the inconsistency in relationships between diversity and ecosystem functions underscores the need for additional studies to disentangle the factors confounding this interconnection in soil systems [13–16].

A major contributing factor to variation in microbial communities is the physical structure of the soil, which is a heterogeneous matrix composed of minerals, organic matter, and pore spaces that provide a multitude of habitats for microbes [17]. The soil matrix contains unique environments at a sub-micron to millimeter range that cannot be represented by sampling soil in bulk. These are the length scales more ecologically relevant than bulk samples for soil microorganisms and their functional contribution to ecosystems [17–20]. All soils are primarily comprised of microaggregates—complexes defined as less than 250 μm in diameter, but larger than 53 μm—and composed of minerals, carbonates, and other particles bound tightly together [21]. In many soils, microacroaggregates are further enmeshed by organic material such as bacterial polysaccharides, fungal hyphae, and plant roots to form macroaggregates (>250 μm) [21, 22]. Aggregates differ in their physical and chemical properties from both bulk soil as well as from each other by size [23]. These differences in carbon (C) pools—particulate organic matter, mineral-associated organic matter, dissolved organic carbon—together with variations in other nutrient contents [24, 25] and pore size distribution that influences water and gases diffusion [26] create microhabitats that support distinct microbial communities and metabolic requirements [18, 27, 28].

Previous studies have shown that soil aggregates of different size fractions harbor distinct bacterial [24, 29–31] and fungal communities [31, 32] and have different rates of extracellular enzyme activity [33–36]. Yet, patterns in the abundance of specific taxa, diversity estimates, and enzymatic activities among different sizes have insofar been difficult to generalize. Many studies have focused solely on characterizing taxonomic composition or measuring enzymatic activities in aggregates, whereas the composition of functional genes and their connection with specific taxa have had limited attention [37]. To our knowledge, only one study has considered the functional potential inferred by metagenomic-assembled genomes (MAGs) at the scale of soil aggregates [38]. Building on known differences in structure and composition across soil aggregate fractions, we tested the hypothesis that large and small macroaggregates provide a broader range of physical and chemical microhabitats—such as varied pore networks, organic matter inputs, and redox conditions—than microaggregates and silt and clay fractions. These diverse resource environments are expected to foster functionally distinct microbial communities, potentially supporting a wider array of metabolic processes and contributing to spatial compartmentalization of biogeochemical functions within the soil matrix. In doing so, we performed aggregate size separation from agricultural soils and integrated two high throughput approaches, metagenomic sequencing and metabolomics, in aggregates of different sizes. Metabolic reconstruction of metagenome-assembled genomes also indicated that distinct genera of ammonia-oxidizing archaea may occupy different niches within the same aggregate habitat.

## Materials and methods

### Long-term field trial and soil sampling

Soils used in this study were collected from the Russell Ranch Sustainable Agricultural Research Facility, University of California, Davis (UC Davis), USA (38°32′47″N, 121°52′28″W). A field experiment was initiated in May 2012 to investigate the long-term impacts of different soil amendments on soil carbon storage, microbial communities, and crop production in a Mediterranean agricultural ecosystem [39]. The cropping system consisted of a 2-year rotation of processing tomatoes (*Lycopersicon esculentum* Mill.) and corn (*Zea mays* L.) managed using practices and equipment similar to those used by local commercial growers. Two fertility management systems were tested: mineral fertilizer or poultry manure compost, for which the concentrations applied were scaled to have equivalent total N inputs [40]. Because biochar has received considerable attention as a soil amendment that may increase carbon pools, one application of biochar derived from walnut shells was added at the start of the experiment, resulting in four treatments: (i) mineral fertilizer without biochar, (ii) mineral fertilizer with biochar, (iii) compost without biochar, and (iv) compost with biochar. At the beginning of the planting season, the total N equivalent was applied as either: (i) synthetic fertilizer containing 27.6 kg N ha^−1^ urea-ammonium-nitrate 32, 36.2 kg P ha^−1^ as phosphorus pentoxide, 17.2 kg K ha^−1^ as potassium oxide, and 1.7 kg ha^−1^ of zinc chelate, or (ii) poultry manure compost (8.97 Mg ha^−1^ applied yearly). Specific details on the management, amendments, and soil type have been described previously [39]. The experiment was arranged in a randomized block design with four replicate plots per treatment. In March 2018 before planting, three soil samples per plot were collected from bare soils at a depth of 15 cm using a soil knife, combined into a representative sample (four for each of the field treatments), and transported on ice to the laboratory for subsequent analysis.

### Soil aggregate sieving

Freshly collected bulk soil was first sieved through an 8 mm mesh. Larger clumps were gently broken apart by hand, following the soil’s natural tendency to split along existing cracks or weaker areas. Aggregates were then isolated from bulk soil using the wet sieving method as described previously [40, 41]. Briefly, 50 g of the moist, 8 mm sieved soil was submerged in deionized water on top of a 2000 μm sieve for 5 min. The sieve was moved up and down (~ 3 cm) for 2 min at a rate of 50 repetitions per min [41, 42]. The soil and water passing through the sieve were transferred by gently rinsing the material with deionized water onto the next smaller size sieve, and the same procedure was repeated. Three sieve sizes (2000 μm, 250 μm, and 53 μm) were used to generate four fractions: (i) large macroaggregates (2000–8000 μm), (ii) small macroaggregates (250–2000 μm), (iii) microaggregates (53–250 μm), and (iv) silt and clay (<53 μm). For each fraction, subsamples of the aggregates that were retained on each sieve were collected in sterile 50 ml polypropylene tubes and immediately stored at −80°C until DNA extraction. A total of 16 samples were collected for each of the four aggregate size fractions, resulting in 64 samples.

### Soil DNA extraction and metagenomic sequencing

DNA from each soil aggregate size fraction was extracted in duplicate using the DNAeasy Powerlyzer PowerSoil Kit (Qiagen, Germantown, MD, USA) using a vortex adaptor according to the manufacturer’s instructions. The duplicate extractions of the same soil sample were pooled, and the DNA was quantified using the Qubit High-Sensitivity dsDNA Assay Kit (Life Technologies, Carlsbad, CA, USA) and its quality was determined by gel electrophoresis on a 1% agarose gel. Libraries were prepared using an insert size range of 250–400 bp and sequenced using the Illumina NovaSeq platform (S4 flow cell, paired-end 150 bp). Both library preparation and sequencing were performed at the UC Davis DNA Technologies and Expression Analysis Core Facility (Davis, CA). A total of 1.926 Tbp of sequencing was obtained to reach an average depth of 30.09 Gbp per sample.

### Microbial taxonomic profile

Trimmomatic (v.0.39) [43] was used to remove sequencing adaptors and quality-trim the raw reads using a sliding window of 4 bp, minimum average quality of 30, and a minimum length of 50 bp. To obtain a community-wide taxonomic profile, Kraken2 (v.2.1.2) [44] was used to classify the trimmed reads against a pre-built database of bacteria, archaea, and viruses downloaded from the National Center for Biotechnology Information (NCBI) GenBank [45]. We also used SingleM [46] to estimate prokaryotic community composition (Supplementary Material).

### Functional gene profile

The quality-trimmed reads were assembled into contigs using Megahit (v.1.0.6) [47] with a minimum contig length of 1000 bp. Prodigal (v.2.6.3) [48] was used to identify open reading frames from the contigs and the protein-coding sequences were annotated using KOfamscan (v.1.3.0) [49], which assigns KEGG orthologs (KO) to query genes using Hidden Markov Model (HMM) profiles with predefined score thresholds from the Kyoto Encyclopedia of Genes and Genomes (KEGG) database [50]. To quantify functional genes, the raw reads were mapped back to the assembled contigs using Bowtie2 (v.2.4.5) [51], and the mapping files were used to generate gene counts using HTSeq (v.1.99.2) [52]. A reproducible workflow is available at https://github.com/cErikson/GeneLab_Shotgun_Metagenomics_Pipeline.

### Metagenome-assembled genomes

To infer taxonomy from the potential functions assigned at the community level, the assembled contigs were binned using MaxBin2 (v.2.2.7) [53], which recovers genomes from metagenomes based on tetranucleotide frequencies and contig coverages. MaxBin2 was run using the BAM files generated from Bowtie2 that were converted to per-contig coverage information with the “pileup.sh” script in BBMap (v.38.18) [54]. The resulting bins were checked for completeness and quality with CheckM (v.1.1.3) [55]. Only bins with >50% completion and <10% contamination as determined by CheckM were considered for downstream analysis. These criteria, established by Bowers *et al.* [56], define the selected bins as MAGs. The taxonomy of each MAG was assigned using GTDB-Tk (v.2.0.0) [57] against the Genome Taxonomy Database (GTDB) release 07-RS207. The phylogenetic tree was constructed using FastTree2 (v. 2.1.10) based on concatenated alignments of 49 universal single-copy marker genes and visualized in TreeViewer [58]. DRAM (v.0.1.2) was used for the MAGs functional annotation. CoverM (v.0.6.1) (https://github.com/wwood/CoverM) was used to determine the relative abundance of the MAGs classified as archaea after dereplicating them at an average nucleotide identity (ANI) of 99% [59]. Metabolic pathways were constructed from the K number assignments using KEGG mapper [60].

### Quantitative PCR, untargeted metabolomics, and enzyme assays

Information on primers, amplification conditions, and sensitivity of the quantitative PCR (qPCR) assay, metabolomic detection method, and enzymatic assays can be found in Supplementary Material.

### Statistical analysis

All statistical analyses were performed in R (v.3.6.2). For all analyses, a value of *P* < .05 was considered statistically significant. No differences in the taxonomic and functional gene profile were detected from the addition of biochar to the mineral fertilizer and manure compost treatments, which was consistent with our previous report on its limited effect on soil C and bacterial community composition [40]. Therefore, the replicates for biochar addition for each fertilizer treatment (mineral fertilizer vs. manure compost) were combined for downstream analyses. Bray–Curtis distance matrices were calculated for the taxonomic, functional gene, and metabolite community profiles for non-metric multidimensional scaling (NMDS) ordination analysis to visualize overall differences in composition between aggregate size fractions. Differences were tested by permutational multivariate analysis of variance (PERMANOVA) running 999 permutations with the “adonis” function in the “vegan” package [61] using aggregate size, fertilizer treatment, and their interaction as predictor variables. The “edgeR” package [62] was used to estimate dispersions, transform reads to log_2_-counts per million (CPM), and perform statistical tests to identify differentially abundant taxa and functional genes between the large and small macroaggregates with the microaggregates and silt and clay. Differentially abundant taxa and functional genes were visualized using heatmaps produced with the “pheatmap” package [63]. The “clusterProfiler” package [64] was used to identify enriched KEGG pathways based on differentially abundant KO terms. Differential abundance analysis of the metabolomics data was performed using the “limma” package [65] after normalizing samples by the sum of all peak heights for all identified metabolites. Metabolite data are presented as log_10_-transformed peak heights. All other univariate data were first tested for assumptions of normality and homogeneity of variance before comparison using a one-way analysis of variance (ANOVA) test and Tukey honestly significant difference post-hoc test to identify significant differences between aggregate sizes. Data that failed to meet assumptions for ANOVA were compared by using a non-parametric Kruskal-Wallis test followed by a Dunn’s post hoc test.

## Results

### Soil microbial community structure at the aggregate level

**Figure 1:**
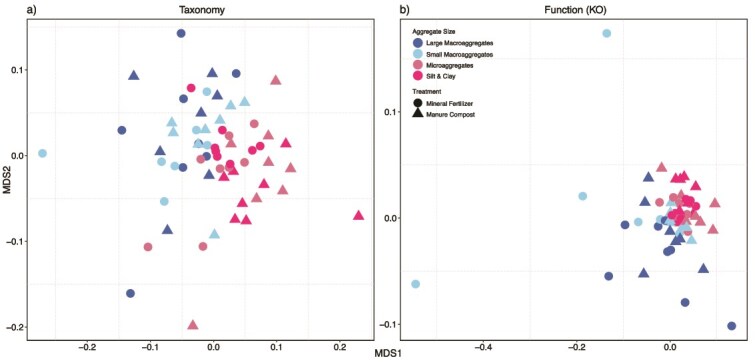
Microbial taxonomic and functional community structure across soil aggregate size fractions. NMDS plots based on Bray–Curtis dissimilarities calculated from (a) Kraken 2 species-level taxonomic profiles and (b) KEGG Orthology functional profiles. Samples are colored by aggregate size, and shapes indicate fertilizer treatments. Ordination stress values were 0.222 for the taxonomic profile and 0.091 for the functional profile.

**Figure 2:**
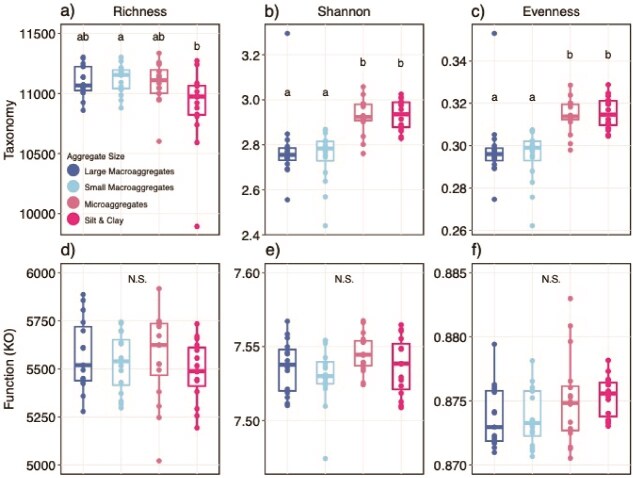
Alpha diversity of microbial taxonomic and functional profiles across soil aggregate size fractions. (a–c) Taxonomic alpha diversity based on Kraken2 species-level profiles and (d–f) functional alpha diversity based on KEGG Orthology profiles, including richness, Shannon diversity, and Pielou’s evenness. Samples are colored by aggregate size fraction. Different letters indicate statistically significant differences (*P* < .05) among aggregate size fractions.

After quality-filtering, the total number of metagenomic reads per sample ranged from 27 974 166 to 106 652 794 with an average of 84 019 069 (Supplementary Table ST1). Overall, the microbial community composition at the species level was significantly different (*P* < .001) by aggregate size (Table 1a). A small but significant difference in community composition (*P* < .001) was found across all samples by agricultural management treatment, mainly mineral fertilizer vs. compost application, but not from the interaction between aggregate size and treatment (Table 1a). These differences are most apparent with the separation of microbial communities by aggregate size observed in the NMDS ordination (Fig. 1a, Supplementary Fig. SF2c). Among the different aggregate size fractions, the silt and clay had a lower richness compared to the small macroaggregates (Fig. 2a). However, alpha diversity was higher (*P* < .05) in the microaggregates and silt and clay than in the large and small macroaggregates based on the Shannon index (Fig. 2b), and these differences corresponded with a higher evenness (*P* < .05) of the microbial community in the microaggregates and silt and clay than in the two macroaggregate fractions (Fig. 2c). Similar trends were observed using SingleM, with microaggregates and silt and clay showing a tendency toward higher diversity and evenness relative to the macroaggregate fractions (Supplementary Fig. SF2a). Across all samples, the taxonomic alpha diversity was higher (*P* < .05) in the manure compost treatment compared to the mineral fertilizer treatment (Supplementary Fig. SF1a–c). No significant differences in functional gene alpha diversity were detected among aggregate size fractions (Fig. 2d–f). However, KO richness values were uniformly high across samples, suggesting saturation of the detectable KEGG Orthology space in these deeply sequenced metagenomes. Thus, the similarity in functional richness should not be interpreted as evidence that the functional repertoire was biologically stable across aggregate fractions, but rather as a broad detection of KO-annotated functions across samples. In contrast, functional beta diversity revealed significant differences among aggregate-size fractions, indicating that the size of an aggregate influenced the relative abundance and composition of functional genes rather than the overall number of detected KO terms (Table 1b, Fig. 1b).

**Table 1:** Permutational multivariate analysis of variance (PERMANOVA) of microbial taxonomic composition (a) and functional composition (b) across soil aggregates, followed by pairwise PERMANOVA comparisons between aggregate sizes.

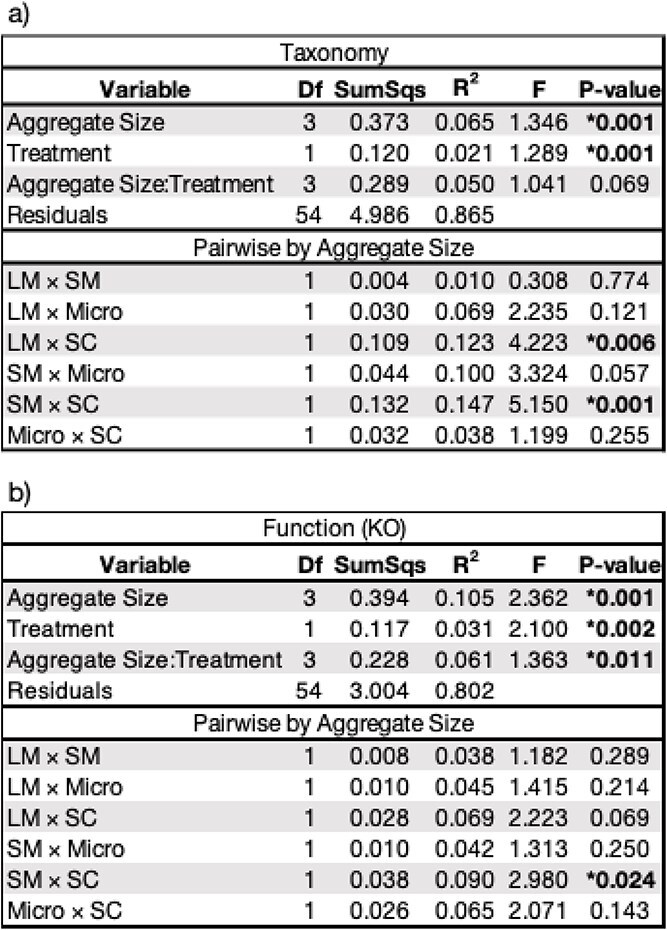

**Figure 3:**
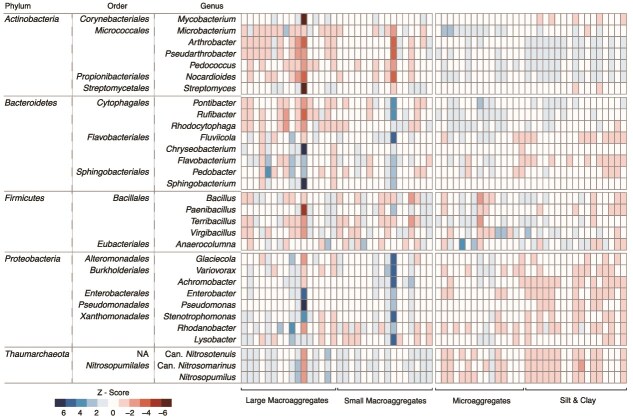
Heatmap showing taxa at the genus level that were differentially abundant (*P* < .05) between the large and small macroaggregates and microaggregates and silt and clay. Corresponding taxonomic ranks for order and phylum are given for each genus.

Differences in the abundance of taxonomic groups among different aggregate sizes followed phylum- and order-specific patterns. First, the microaggregates and silt and clay harbored higher abundances (*P* < .05) of several genera within the phyla *Actinobacteria* and *Firmicutes* than did large and small macroaggregates (Fig. 3). In contrast, eight genera within the *Proteobacteria* and three genera within the archaeal phylum *Thaumarcheota* were enriched (*P* < .05) in large and small macroaggregates than microaggregates and silt and clay (Fig. 3). The genera *Pontibacter, Rufibacter*, and *Rhodocytophaga* from the order *Cytophagales,* phylum *Bacteroidetes,* were found in higher abundances (*P* < .05) in the microaggregates and silt and clay, whereas *Fluviicola, Chryseobacterium*, and *Flavobacterium* within the *Flavobacteriales* and *Pedobacter* and *Sphingobacterium* in the *Sphingobacteriales* were enriched (*P* < .05) in the large and small macroaggregates (Fig. 3). Across all samples, the manure compost treatment had a higher abundance (*P* < .05) of *Pseudomonas, Chryseobacterium, Rhodococcus, Sphingobacterium*, and *Pedobacter*, while the mineral fertilizer treatment had a higher abundance of *Rhodanobacter, Pantoea*, and *Nitrosospira* (Supplementary Table ST3a). Using the SingleM approach, we observed consistent shifts in the relative abundance of key phyla between aggregate size groups. *Thermoproteota* (*Thaumarcheota* in GTDB) was slightly enriched in macroaggregates (LM + SM, 6.75%) compared to microaggregates (Micro+SC, 5.17%). In contrast, both *Pseudomonadota* (*Proteobacteria* in GTDB) and *Actinomycetota* (*Actinobacteria* in GTDB) were relatively more abundant in microaggregates (14.50% and 47.31, respectively) than in macroaggregates (13.66% and 45.12%, respectively; Supplementary Fig. SF2c). These results indicated that soil aggregates of different size fractions harbor microbial communities distinct in composition and diversity, with the differences in microbial groups following broad taxonomic levels.

### Functional gene profile and associated taxa in soil aggregates

We assembled metagenomic reads, identified genes from the contigs, and annotated them using the KEGG database [48] to obtain a community-wide functional gene profile in aggregates. The microbial functional gene composition was significantly different (*P* < .001) by aggregate size, agricultural management treatment, and the interaction between both variables (Table 1b). Aggregate size had the largest effect in the PERMANOVA model, explaining 10.5% of the variation in functional gene composition (Table 1b) for which an effect of separation of samples by aggregate size was observed in the NMDS plot (Fig. 1b). No differences in functional gene alpha diversity by aggregate size were detected (Fig. 2d–f). Across all size fractions, the manure compost treatment had higher functional gene richness and Shannon index but not evenness compared to the mineral fertilizer treatment (Supplementary Fig. SF1d–f).

**Figure 4:**
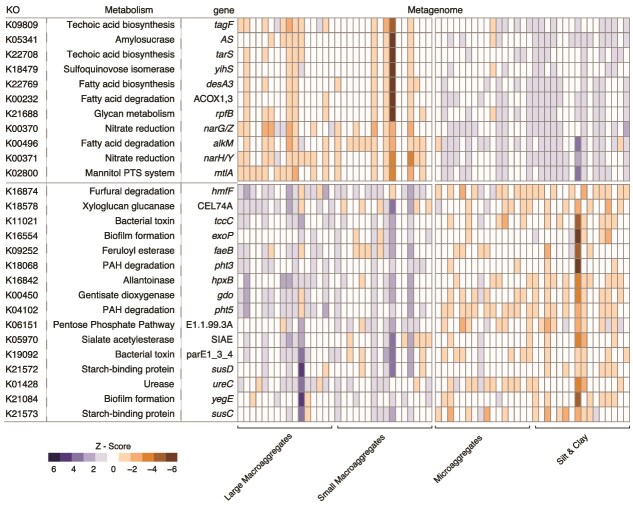
Heatmaps showing the a) functional genes in soil aggregates that were differentially abundant (*P* < .05) between the large and small macroaggregates and microaggregates and silt and clay. Relevant KEGG pathways, modules, or enzyme names are given with the K numbers for each gene.

Out of the 144 differentially abundant genes identified among aggregate sizes (Supplementary Table ST4), 27 genes (~19%) were associated with metabolic functions involved with biogeochemical C and N cycling, microbial substrate assimilation, biosynthesis, and other intracellular processes (Fig. 4). The microaggregate and silt and clay fractions were enriched in genes for nitrate reduction (*narG/Z, narH/Y*), mannitol transport (*mtlA*), synthesis of microbial polymers (AS) and co-polymers (*tagF, tarS*), and degradation of fatty acids (*ACOX1*,3, *alkM*), glycans (*rpfB*), and sulfoquinovose (*yihS*) (Fig. 4). On the other hand, the large and small macroaggregates contained a higher abundance of genes for waste N recycling, including allantoinase (*hpxB*) and urease (*ureC*); and degradation of potential plant-derived compounds, including starch (*susC, susD*), xyloglucan (*cel74A*), xylan (*faeB*), sialate (*siae*), furfural (*hmfF*), gentisate (*gdo*), and other polycyclic aromatic hydrocarbons (*pht3, pht5*) (Fig. 4). Genes encoding for bacterial toxins (*tccC*, parE1_3_4) and biofilm formation (*exoP, yegE*) were also enriched in the large and small macroaggregates. The manure compost treatment was significantly enriched in genes for fatty acid degradation, amino acid metabolism, and transporters of various compounds compared to the mineral fertilizer treatment (Supplementary Table ST3b). Based on the differentially abundant KO terms, the KEGG pathways for beta-lactam resistance, arginine biosynthesis, and two-component systems were enriched in the large and small macroaggregates, whereas pathways for arabinogalactan biosynthesis and biosynthesis of unsaturated fatty acids were enriched the microaggregates and silt and clay (Supplementary Fig. SF3).

### Soil aggregate metabolomes and enzyme activities

**Figure 5:**
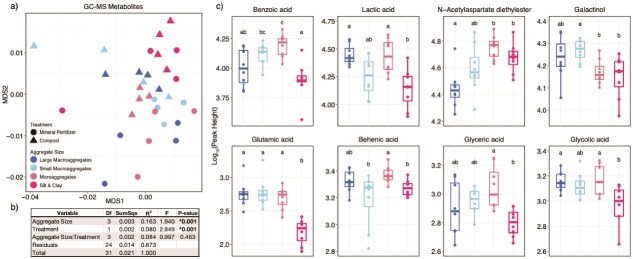
(a) NMDS plot based on the Bray–Curtis distance of the metabolome of soil aggregates. Metabolites were identified by GC–MS, and the ordination stress value was 0.171. (b) Permutational multivariate analysis of variance (PERMANOVA) results for the metabolome and (c) the top 8 identified metabolites that were differentially abundant based on log fold change. Samples in the NMDS are colored by aggregate size, and shapes correspond with different fertilizer treatments. Different letters in the boxplots represent significant differences (*P* < .05) between aggregate size fractions.

To determine whether differences in functional potential observed by metagenomics translated into metabolic output differences, we performed metabolomics on each aggregate size fraction. Overall, the metabolomics profile was different by aggregate size (*P* < .001) and fertilizer treatment (*P* < .001), but not from their interaction, with the greatest effect of separation in the metabolites observed by aggregate size (Fig. 5a and b). Out of 173 detectable metabolites, 96 were identified (55.5%). The top 8 identifiable metabolites that were differentially abundant (*P* < .05) based on log-fold change are shown in Fig. 5c and the remaining 6 metabolites with statistical significance (*P* < .05) are shown in Supplementary Fig. SF4. Notable compounds that were different by aggregate size included lactic acid, galactinol, glutamic acid, glycolic acid, and adenosine (Fig. 5c), which were enriched in the macroaggregates; and N-acetyl aspartate diethylester and putrescine (Fig. 5c), which were enriched in the microaggregates and silt and clay. The manure compost treatment was higher in isomaltose and beta-sitosterol, whereas the mineral fertilizer treatment was higher in palmitic acid across all samples (Supplementary Table ST3c).

No differences in overall enzyme activities by aggregate size or agricultural management treatment were observed (Supplementary Fig. SF5a and b). No significant differences in the activity of specific enzymes were detected between any of the aggregate size fractions except for phosphatase, which had higher activity (*P* < .05) in the silt and clay compared to the small macroaggregates (Supplementary Fig. SF5c).

### Abundance and potential function of archaea MAGs

A total of 152 MAGs (with >50% completeness and < 10% contamination) were retrieved from different aggregate samples. Of this total, 65 belonged to the Domain *Bacteria* and 87 were identified as members of the *Archaea* (Supplementary Fig. SF6). The bacterial MAGs were represented by various lineages within the phyla *Actinobacteriota* (*n* = 19), *Acidobacteriota* (*n* = 8), *Chloroflexota* (*n* = 8), *Firmicutes* (*n* = 15), *Proteobacteria* (*n* = 6), *Desulfobacterota* (*n* = 3), *Gemmatimonadota* (*n* = 4), and *Nitrospirota* (*n* = 2), whereas all the 87 archaeal MAGs were classified as belonging to the order *Nitrososphaerales* within the phylum *Thermoproteota* (formerly *Thaumarchaeota*, [66]) (*n* = 87, Supplementary Fig. SF6 and Supplementary Table ST5).

Recently, it was reported that macroaggregates contained higher abundances of ammonia-oxidizing archaea (AOA), within the phylum *Thermoproteota* [67] and nitrification rates [68]. Thus, we further analyzed our archaea MAGs to characterize their phylogeny, evaluate their ecological distribution, and determine their potential functions in aggregates. In total, 38 archaeal MAGs were retrieved from macroaggregates and 49 from microaggregates. Among these, 54 MAGs demonstrated completeness greater than 90%, thus classified as near-complete or high-quality genomes [54] (Supplementary Table ST5), and were subsequently selected for detailed analysis. Phylogenetic analysis revealed that the archaea MAGs represented two distinct genera within the family *Nitrososphaeraceae*. The first group, represented by 8 MAGs, was classified as a species within the genus *Nitrososphaera,* while the other 38 MAGs formed a cluster within the as-yet uncultivated genus TH1177 (Supplementary Fig. SF7).

Next, we performed MAG sequence dereplication to estimate their abundances and observed that both lineages had higher relative abundances in macroaggregates. The representative MAG for the *Nitrososphaeraceae* TH1177 (A46_maxbin.003) was observed to be more abundant (*P* < .05) in large macroaggregates than in microaggregate and silt and clay fractions (Supplementary Fig. SF8a), whereas the MAG representing the *Nitrososphaera* species (A49_maxbin.001) was more abundant in both large and small macroaggregates compared to the two smallest aggregate size fractions (Supplementary Fig. SF8b). These findings were confirmed by qPCR targeting the *amoA* gene in archaea, where AOA copy numbers were higher (*P* < .05) in the large and small macroaggregates compared to the silt and clay (Supplementary Fig. SF8c) and significantly correlated with the relative abundances of both archaea MAGs (*P* < 8.1 × 10^−7^, *P* < 1.1 × 10^−5^, Supplementary Fig. SF8d and e, respectively).

**Figure 6:**
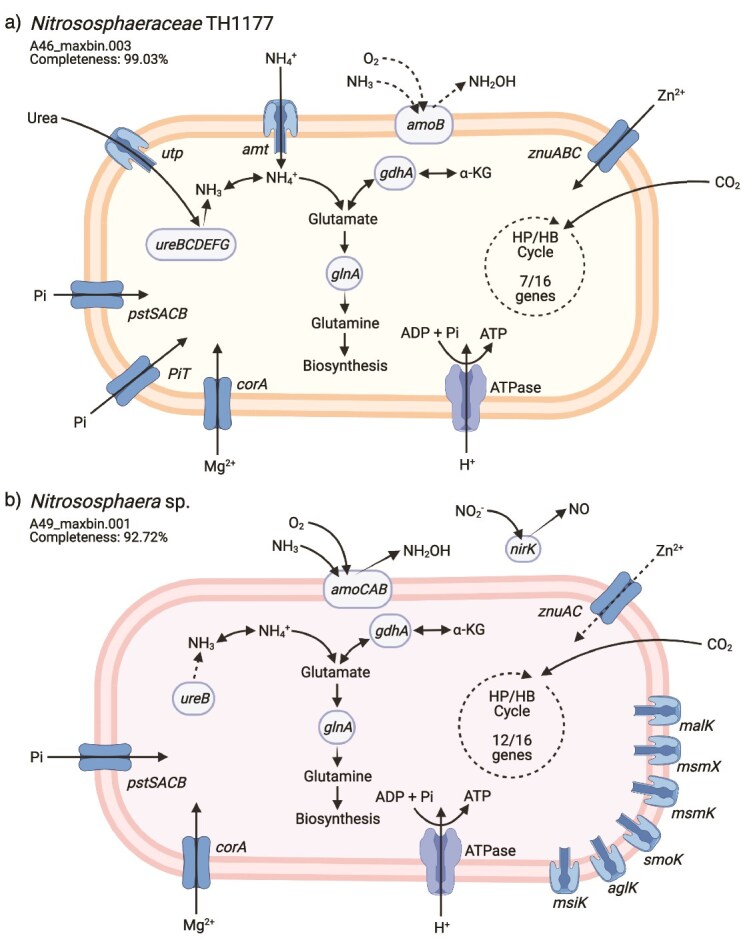
Cell metabolism diagrams constructed from the MAGs of (a) A46_maxbin.003, which was classified as a species within the genus *Nitrososphaeraceae* TH1177, and (b) A49_maxbin.001, which was classified as a species within the genus *Nitrososphaera*. Dashed lines represent putative pathways due to incomplete operons or missing genes.

Pathway reconstruction revealed differences in the genomic composition between the two MAGs (Fig. 6). A46_maxbin.003 possessed a gene for one subunit of ammonia monooxygenase (*amoB*), a gene for an ammonium transporter (*amt*), a complete gene cluster for urease (*ureBCDEFG*) and urea transporter (*utp*), zinc transporter (*znuABC*), both high and low affinity phosphate transporters (*pstSACB* & *PiT*) and genes for CO_2_ fixation (Fig. 6a). On the other hand, A49_maxbin.001 contained a complete gene cluster for ammonia monooxygenase (*amoCAB*), nitrite reductase (*nirK*), an incomplete zinc transporter (*znuAC*), and only the high affinity phosphate transporter (*pstSACB*) (Fig. 6b). Furthermore, compared to A46_maxbin.003, A49_maxbin.001 possessed genes for several ABC-type transporters of multiple saccharides and polyols (*malK, msmX, msmK, smoK, aglK, msiK*) as well as a higher number of genes involved in the CO_2_-fixing hydroxypropionate/hydroxybutyrate (HP/HB) cycle (Fig. 6b).

## Discussion

Differences in the physicochemical properties within macro- and microaggregates make them unique habitats for distinct communities of microorganisms within the soil environment [20, 25–28]. We indeed found significant differences in the microbial taxonomic- and functional gene composition and detected metabolites among aggregate sizes. Our results suggested the different aggregate size fractions as resource-rich or resource-poor microhabitats. Notably, we found higher alpha diversity (Shannon index) in microaggregates and silt and clay fractions than in large and small macroaggregates. While microaggregates and silt and clay harbored higher abundances of members within the phyla *Actinobacteria* and *Firmicutes*, large and small macroaggregates had higher abundances of *Thaumarchaeota* and *Proteobacteria*. These findings corroborate results from our previous study using the 16S rRNA gene to taxonomically characterize aggregates of the same experimental plots, where higher proportions of the *Actinobacteria* orders *Micrococcales, Streptomycetales*, and *Propionibacteriales* were observed in microaggregates and silt and clay fractions, while a higher proportion of *Thaumarchaeota* order *Nitrososphaerales* was detected in large and small macroaggregates [40]. Our results also show a significant effect of the agricultural management treatment with increased genomic diversity with application of compost in comparison to the mineral fertilizer. The application of biochar as a C amendment did not have an effect on genomic diversity. We attribute this result to the timing of the biochar application at the start of the experiment and its recalcitrant nature, fading any direct effect on soil microbial communities, while compost and mineral fertilizer applications occurred on a yearly basis.

While molecular characterizations of microbial communities in aggregates based on amplicon sequencing or clone libraries have been conducted in soils under agricultural management [17, 24, 29–32], only two studies found similar patterns in which microbial groups inhabited different size fractions. For instance, Mummey *et al.* [69] found that the *Actinobacteria* were most abundant in microaggregates in comparison to macroaggregates. Similarly, Bach *et al.* [31] found higher relative abundances and alpha diversity of several *Actinobacteria* orders in microaggregates [31, 32]. Higher abundances of α-*Proteobacteria* [35] and ammonia-oxidizing archaea [67, 68] have also been found in macroaggregates using qPCR. However, others observed contrasting patterns [24, 30], where most *Actinobacteria* groups (except *Rubrobacteriales*) were more abundant in the macroaggregates than smaller aggregates. We surmise differences among studies may be due to two main factors: First, different methodological approaches were used to perform size fractionation of aggregates from the bulk soil [70]. Soil aggregates are often collected using either dry sieving, which involves shaking bulk soil on top of a stack of sieves; or wet sieving, which submerges the bulk soil in water followed by repeated vertical strokes on top of a sieve while still immersed [42]. These very different sieving methods would be expected to differentially impact microbial extracellular enzyme activity [34] and bacterial and fungal community composition in aggregates [71]. Previously, we have confirmed the above findings for the same soils, indicating a differential response between prokaryotic and fungal communities as a result of the soil aggregate isolation method [41]. Second, observed differences in taxa distribution and species diversity may reflect local adaptation of resident microbes to variations in aggregate formation and reveal a direct influence of soil texture, agricultural management, or even microbial processes experienced during community assembly [72].

Next, we evaluated functional gene differences among aggregate size fractions. Aggregates are spatially structured environments [17, 23] with macroaggregates being composed of microaggregates bound together by various organic compounds, including plant roots, fungal hyphae, and bacterial exopolysaccharides [73, 74]. As macroaggregates are formed, this pool of organic matter can become occluded within their interiors [26], protected from degradation by externally located microbes (i.e. on aggregate surfaces) but can serve as a nutrient source for those microbial communities within [20]. Our results strongly support the hypothesis that large and small macroaggregates provide an increased number of resources in comparison to microaggregates and silt and clay. Two lines of evidence support our findings: first, the functional gene composition of the microbial community observed in large and small macroaggregates was associated with the degradation of plant-derived compounds. We found a significant enrichment of genes for the breakdown of xylan (*faeB*), xyloglucan (*cel74A*), furfural (*hmfF*), and gentisate (*gdo*). Feruloyl esterase, encoded by *faeB*, cleaves ester linkages in xylan to release aromatic acids [75], whereas xyloglucan exo-beta-1, 4-glucanase (*cel74A*) hydrolyses xyloglucan to release oligosaccharides [76]. Furfural is formed naturally by the dehydration of xylose, a sugar that is abundant in the hemicellulose fraction of lignocellulosic biomass [77], while gentisate (2, 5-dihydroxybenzoate) is a phenolic acid that is widely distributed as a secondary plant product [78]. Supporting this line of evidence, our earlier study at same field site determined higher concentrations of total C in small aggregates than in microaggregates and silt and clay [41]. Second, our metabolite measurements indicated a higher abundance of compounds involved in microbial energy metabolism in macroaggregates, perhaps indicating a higher availability of nutrients than in microaggregates and silt and clay fractions, where increased concentrations of putrescine and N-acetyl aspartate diethylester were observed. We acknowledge a limitation of the wet sieving protocol as it inherently exposes the microaggregate and silt and clay fractions to a higher degree of water contact due to their disproportionately larger surface area-to-volume ratios compared to macroaggregates. Consequently, the lower observed abundances of highly soluble metabolites, such as glutamic acid, lactic acid, and adenosine, in these smallest fractions may be partially or entirely driven by preferential leaching during the isolation process rather than biological differences. Nevertheless, the overarching separation of the metabolome remained significantly attributed to aggregate size (*P* < .001), suggesting broad metabolic differences beyond just the most soluble compounds. Other studies have shown higher concentrations of organic carbon in macroaggregates than in microaggregates and silt and clay [24], and that macroaggregates preferentially accumulate phenolic, carboxyl, and methoxyl/N-alkyl C compounds from agricultural inputs during formation from microaggregates [25]. Taken together, the above results suggest that macroaggregates are more resource-rich habitats for microbes than microaggregates.

Acknowledging that increased concentrations of microbially-available nutrients may result in elevated competition or facilitation between microbial species, we analyzed genomic traits related to competition and survival. There is evidence of an increased abundance of genes for bacterial toxin formation, allantoinase, urease, and biofilm formation in macroaggregates. Toxin production is a strategy used by some lineages of bacteria to defend against predation by insects or soil microfauna [79]. A higher abundance of the *ureC* gene in macroaggregates has been reported previously [80] and, together with the gene for allantoinase (*hpxB*), is an indicator that some microbial groups may benefit from recycling waste N or other N-containing metabolites, such as glutamic acid, identified by our metabolomics approach. Bacteria can form biofilms to stabilize conditions in their immediate environment and increase the efficiency of resource acquisition [81]. Bacteria and fungi preferentially occupy pores measuring between 1 μm and 1 mm in size, and biofilms can form in the large pores prevalent within macroaggregates to take advantage of moisture, oxygen, and nutrients diffusing from the soil matrix [82]. This finding contrasts with our detection of enriched genes for dissimilatory nitrate reduction (*narG/Z, narH/Y*) in microaggregates and silt and clay, which have much smaller pores, limiting gas diffusion into their interiors particularly when saturated with water [23]. This result suggests that more prokaryotes in the smallest aggregate size fractions have the capability to use alternate terminal electron acceptors under anoxic conditions.

While evaluating microbial genomic differences among aggregate size fractions, genes related to the synthesis and degradation of fatty acids and other polymers of microbial origin were observed to be more abundant in microaggregates and silt and clay than in macroaggregates. Microaggregates often contain little to no plant debris [18, 21, 22], and the organic matter that is present is typically associated with phyllosilicates, metal oxides, and other minerals that restrict microbes from access [23]. Therefore, the degradation of microbial biomass - including microbial solutes, extracellular polymeric substances, and cell wall remnants - may be the only sources of nutrients available for resident microbes. The genes identified in different size fractions provided support for this hypothesis. For example, a gene for a mannitol phosphotransferase system (*mtlA*) was significantly enriched in microaggregates and silt and clay than in other fractions. Mannitol is a widely distributed polyol produced by fungi as a major storage compound and by bacteria as a solute in response to osmolarity stress [83]. The increased abundance of *mtlA*, together with other genes for the degradation of microbial glycans and aliphatic hydrocarbons, suggests that microorganisms within the microaggregates and silt and clay may favor assimilation of microbially-derived products for survival. Recent studies have shown that most of the stable organic matter formed in soil is derived from microbial necromass [84, 85] and that the mineral-associated organic matter in microaggregates is predominantly microbial in origin [86]. This provides evidence that microbial biomass in the microaggregates and silt and clay can be used by other microbes as a nutrient source. However, whether the resident microbes that are “entombed” within the microaggregates are active in recycling biomass has not yet been confirmed.

Not only did aggregates vary in genomic composition of their microbial communities but also, in their metabolites. Notably, glutamic acid, lactic acid, galactinol, glycolic acid, and adenosine concentrations were more enriched in macroaggregates than in the two smallest aggregate size fractions. These metabolites reflect a higher abundance of resources in macroaggregates that can support microbial growth and energy metabolism, consistent with our hypothesis that a higher variety of resources are present in large and small macroaggregates than in microaggregates and silt and clay. We did not, however, detect any differences in enzymatic activities for six of seven enzymes that we assayed; only phosphatase activity was higher in silt and clay than other aggregate fractions. The significant result for phosphatase could be related to the slow diffusion of phosphate in soils in comparison to substrates used for the other enzymes. Therefore, microorganisms present in microaggregates might have access to C and N substrates but have to rely on local P sources. Enzyme substrates, such as xylose, sucrose, glucose, and leucine, and their products are consumed and generated rather quickly, masking any significant differences caused by aggregate size or agricultural management treatment.

The coexistence theory, a classical framework in ecology, states that similar species can be stably maintained in proximity as a result of niche partitioning due to resource availability [87]. Observational support has been advanced in animal and plant communities for this theory [88, 89] but is not commonly evaluated for soil microorganisms [90]. Although our study was not designed explicitly to empirically test pairwise coexistence, such as the elegant studies of Estrela *et al.* [91] and Chang *et al.* [92], our MAG analysis of genomic trait variation in soil aggregates provides additional support for this theory. Two >90% completeness MAGs of distinct archaeal genera within the family *Nitrososphaeraceae* were identified in large and small aggregates and their abundances later confirmed by qPCR. The genus *Nitrososphaera* are common in agricultural soils, where synthetic ammonium fertilizer is applied, and known to perform the important process of nitrification [93, 94]. Our genomic results established that these MAGS belong to ammonia-oxidizing archaea (AOA) carrying the canonical genes for CO_2_ fixation, ammonia monooxygenase, and nitrite reductase. Our results corroborate previous studies detecting higher abundances of AOA in macroaggregates than microaggregates based on qPCR [67, 95], but contrast with two other studies that found high abundances in microaggregates [96] or no differences by aggregate size [97]. Notably, a recent study by Wu *et al.* [38] using a similar coverage-based method to estimate the abundance of AOA MAGs found two of their detected *Nitrososphaeraceae* MAGs were more abundant in macroaggregates, and another in microaggregates, indicating potential ecotypic variation [38]. Similarly, our two AOA MAGs appeared to vary in functional potential. Despite being nearly complete (99.03%), the MAG of A46_maxbin.003 (genus TH1177) did not contain a complete operon for ammonia monooxygenase compared to A49_maxbin.001 (genus *Nitrososphaera*), which has a complete *amoCAB* gene cluster in addition to the *nirK* gene. The A46_maxbin.003 MAG, instead, possesses a gene cluster for urease and a urea transporter, which the MAG A49_maxbin.001 lacks. The latter MAG also contains several genes encoding ABC-type transporters of various polysaccharides, oligosaccharides, and polyols which are absent in the genome of A46_maxbin.003. These differences between the two MAGs suggest a potential for the two lineages to use different nutrient sources and thus support complementary metabolisms. In the absence of ammonia, some AOA (including *Nitrososphaera*) can grow on urea as their sole energy source [98, 99], whereas others can couple organic C assimilation with CO_2_ fixation for an energetic advantage. This demonstrates that these two lineages can occupy different niches within the same macroaggregate habitat [100].

## Conclusion

Our study revealed new insights into soil aggregates as distinct microbial habitats within the overall soil matrix. We found that the microbial taxonomic and functional gene composition varied significantly among different aggregate sizes, and our analysis of functional genes, MAGs, and metabolites characterized macroaggregates as environments rich in resources derived from different agricultural management inputs. In other words, the quality of the resources matters to the aggregate as a microbial habitat. On the other hand, the microaggregates and silt and clay represented resource-limited habitats with metabolic pathways largely restricted to the recycling of microbial biomass and anaerobic respiration. Taken together, our results provide evidence that aggregates support different niches—diversified C sources and electron acceptors—for microorganisms to colonize. Therefore, soils with more macroaggregates are in principle expected to harbor a higher diversity of microorganisms and microbially driven ecosystem benefits [101]. Because aggregates are rarely considered in surveys of soil microbial diversity [35], this study demonstrates that a closer examination of the spatial stratification provided by aggregates is needed if one wants to understand species diversity and function at the scale that matters to microorganisms.

## Supplementary Material

Supplementary_material_ycag161

## Data Availability

The raw sequencing data were deposited to the NCBI sequence read archive (SRA) under BioProject PRJNA847587. The MAG files in FASTA format are available on figshare: https://doi.org/10.6084/m9.figshare.28850741.
