# Supplementary material for: Multi-omics resolved integration reveals microbial niche separation in soil aggregates
Source: ISME Commun. 2026 Jun 11;6(1):ycag161. doi: 10.1093/ismeco/ycag161 (PMC13398701; doi:10.1093/ismeco/ycag161)
Supplement: Supplementary_material_ycag161 [file supplementary_material_ycag161.zip › SupplementaryTable_ST5.docx]

**Supplementary Table ST4**. Summary metrics and taxonomic classifications of metagenome assembled genomes (MAGs) used in this study.

| **MAG ID** | **Completion (%)** | **Contamination (%)** | **Heterogeneity (%)** | **Genome Size (Mbp)** | **GC Content (%)** | **GTDB Classification** | | | | | | |
| --- | --- | --- | --- | --- | --- | --- | --- | --- | --- | --- | --- | --- |
|  |  |  |  |  |  | **Domain** | **Phylum** | **Class** | **Order** | **Family** | **Genus** | **Species** |
| A1_maxbin.008 | 99.03 | 8.74 | 8.33 | 3.61 | 28.8 | Archaea | Thermoproteota | Nitrososphaeria | Nitrososphaerales | Nitrososphaeraceae | TH1177 |  |
| A1_maxbin.002 | 69.42 | 5.55 | 41.67 | 1.57 | 50.1 | Archaea | Thermoproteota | Nitrososphaeria | Nitrososphaerales | Nitrososphaeraceae | Nitrososphaera |  |
| A2_maxbin.001 | 92.72 | 4.85 | 0.00 | 1.76 | 29.9 | Archaea | Thermoproteota | Nitrososphaeria | Nitrososphaerales | Nitrososphaeraceae | TH1177 |  |
| A2_maxbin.017 | 64.07 | 2.95 | 25.00 | 2.02 | 55.9 | Bacteria | Proteobacteria | Gammaproteobacteria | JACCYU01 | JACCYU01 |  |  |
| A3_maxbin.012 | 99.03 | 3.88 | 0.00 | 2.54 | 58.1 | Archaea | Thermoproteota | Nitrososphaeria | Nitrososphaerales | Nitrososphaeraceae | TH1177 |  |
| A3_maxbin.004 | 58.73 | 2.90 | 29.41 | 2.39 | 38.5 | Bacteria | Firmicutes | Bacilli | Bacillales | Bacillaceae | Priestia | megaterium |
| A4_maxbin.005 | 99.03 | 4.85 | 0.00 | 3.00 | 28.7 | Archaea | Thermoproteota | Nitrososphaeria | Nitrososphaerales | Nitrososphaeraceae | TH1177 |  |
| A4_maxbin.004 | 69.82 | 7.00 | 55.00 | 1.56 | 40.0 | Bacteria | Firmicutes | Bacilli | Bacillales | Bacillaceae | Priestia | megaterium |
| A4_maxbin.047 | 63.35 | 4.85 | 22.22 | 5.03 | 33.4 | Archaea | Thermoproteota | Nitrososphaeria | Nitrososphaerales | Nitrososphaeraceae | JAFAQB01 |  |
| A4_maxbin.045 | 55.68 | 6.70 | 33.33 | 4.03 | 75.3 | Bacteria | Actinobacteriota |  |  |  |  |  |
| A5_maxbin.007 | 55.17 | 5.17 | 0.00 | 2.57 | 70.6 | Bacteria | Chloroflexota | Limnocylindria | Limnocylindrales | CSP1-4 | SPCO01 |  |
| A5_maxbin.003 | 51.70 | 4.65 | 0.00 | 2.74 | 68.8 | Bacteria | Gemmatimonadota | Gemmatimonadetes | Gemmatimonadales | GWC2-71-9 | JABFSM01 |  |
| A6_maxbin.001 | 99.03 | 5.65 | 27.27 | 1.95 | 50.9 | Archaea | Thermoproteota | Nitrososphaeria | Nitrososphaerales | Nitrososphaeraceae | Nitrososphaera |  |
| A6_maxbin.002 | 85.28 | 6.31 | 0.00 | 1.69 | 30.1 | Archaea | Thermoproteota | Nitrososphaeria | Nitrososphaerales | Nitrososphaeraceae | TH1177 |  |
| A7_maxbin.004 | 99.03 | 2.91 | 0.00 | 2.86 | 28.5 | Archaea | Thermoproteota | Nitrososphaeria | Nitrososphaerales | Nitrososphaeraceae | TH1177 |  |
| A7_maxbin.001 | 60.36 | 0.49 | 100.00 | 1.07 | 50.8 | Archaea | Thermoproteota | Nitrososphaeria | Nitrososphaerales | Nitrososphaeraceae | Nitrososphaera |  |
| A8_maxbin.003 | 93.20 | 2.91 | 0.00 | 2.96 | 28.6 | Archaea | Thermoproteota | Nitrososphaeria | Nitrososphaerales | Nitrososphaeraceae | TH1177 |  |
| A8_maxbin.057 | 73.38 | 7.77 | 18.18 | 4.79 | 33.4 | Archaea | Thermoproteota | Nitrososphaeria | Nitrososphaerales | Nitrososphaeraceae | JAFAQB01 |  |
| A8_maxbin.004 | 65.10 | 2.18 | 18.75 | 2.56 | 38.5 | Bacteria | Firmicutes | Bacilli | Bacillales | Bacillaceae | Priestia | megaterium |
| A8_maxbin.076 | 59.22 | 8.25 | 11.11 | 4.86 | 36.7 | Archaea | Thermoproteota | Nitrososphaeria | Nitrososphaerales | Nitrososphaeraceae | TH5893 |  |
| A9_maxbin.005 | 92.23 | 3.88 | 0.00 | 2.96 | 28.6 | Archaea | Thermoproteota | Nitrososphaeria | Nitrososphaerales | Nitrososphaeraceae | TH1177 |  |
| A9_maxbin.030 | 60.29 | 7.68 | 7.14 | 3.66 | 59.5 | Bacteria | Acidobacteriota | Blastocatellia | RBC074 | RBC074 |  |  |
| A10_maxbin.003 | 99.03 | 6.80 | 30.00 | 3.01 | 28.6 | Archaea | Thermoproteota | Nitrososphaeria | Nitrososphaerales | Nitrososphaeraceae | TH1177 |  |
| A10_maxbin.001 | 98.22 | 3.62 | 42.86 | 1.80 | 51.3 | Archaea | Thermoproteota | Nitrososphaeria | Nitrososphaerales | Nitrososphaeraceae | Nitrososphaera |  |
| A10_maxbin.010 | 52.72 | 5.73 | 12.50 | 2.16 | 68.2 | Bacteria | Actinobacteriota | Acidimicrobiia | IMCC26256 | PALSA-610 |  |  |
| A11_maxbin.005 | 90.29 | 2.91 | 0.00 | 2.57 | 28.4 | Archaea | Thermoproteota | Nitrososphaeria | Nitrososphaerales | Nitrososphaeraceae | TH1177 |  |
| A11_maxbin.043 | 55.17 | 9.48 | 14.29 | 4.22 | 74.9 | Bacteria | Actinobacteriota | Actinomycetia |  |  |  |  |
| A12_maxbin.002 | 87.38 | 3.88 | 0.00 | 2.68 | 28.6 | Archaea | Thermoproteota | Nitrososphaeria | Nitrososphaerales | Nitrososphaeraceae | TH1177 |  |
| A12_maxbin.003 | 55.99 | 5.33 | 11.11 | 3.82 | 51.9 | Archaea | Thermoproteota | Nitrososphaeria | Nitrososphaerales | Nitrososphaeraceae | Nitrososphaera |  |
| A13_maxbin.008 | 99.03 | 6.80 | 0.00 | 3.35 | 28.9 | Archaea | Thermoproteota | Nitrososphaeria | Nitrososphaerales | Nitrososphaeraceae | TH1177 |  |
| A14_maxbin.004 | 99.03 | 3.88 | 0.00 | 2.96 | 28.6 | Archaea | Thermoproteota | Nitrososphaeria | Nitrososphaerales | Nitrososphaeraceae | TH1177 |  |
| A14_maxbin.001 | 95.15 | 5.45 | 62.50 | 1.76 | 51.4 | Archaea | Thermoproteota | Nitrososphaeria | Nitrososphaerales | Nitrososphaeraceae | Nitrososphaera |  |
| A15_maxbin.002 | 99.03 | 4.85 | 0.00 | 3.09 | 28.6 | Archaea | Thermoproteota | Nitrososphaeria | Nitrososphaerales | Nitrososphaeraceae | TH1177 |  |
| A15_maxbin.001 | 87.38 | 6.95 | 50.00 | 1.76 | 51.4 | Archaea | Thermoproteota | Nitrososphaeria | Nitrososphaerales | Nitrososphaeraceae | Nitrososphaera |  |
| A15_maxbin.065 | 60.34 | 8.62 | 28.57 | 5.17 | 74.7 | Bacteria | Actinobacteriota | Actinomycetia |  |  |  |  |
| A15_maxbin.005 | 55.78 | 1.70 | 23.08 | 1.89 | 38.6 | Bacteria | Firmicutes | Bacilli | Bacillales | Bacillaceae | Priestia | megaterium |
| A16_maxbin.010 | 94.17 | 4.85 | 0.00 | 3.09 | 28.7 | Archaea | Thermoproteota | Nitrososphaeria | Nitrososphaerales | Nitrososphaeraceae | TH1177 |  |
| A16_maxbin.001 | 93.13 | 8.78 | 37.50 | 1.79 | 51.8 | Archaea | Thermoproteota | Nitrososphaeria | Nitrososphaerales | Nitrososphaeraceae | Nitrososphaera |  |
| A16_maxbin.008 | 86.97 | 8.70 | 18.52 | 3.77 | 38.5 | Bacteria | Firmicutes | Bacilli | Bacillales | Bacillaceae | Priestia | megaterium |
| A16_maxbin.006 | 58.99 | 7.75 | 20.00 | 3.22 | 69.1 | Bacteria | Gemmatimonadota | Gemmatimonadetes | Gemmatimonadales | GWC2-71-9 | JABFSM01 |  |
| A17_maxbin.008 | 99.03 | 3.88 | 0.00 | 3.01 | 28.5 | Archaea | Thermoproteota | Nitrososphaeria | Nitrososphaerales | Nitrososphaeraceae | TH1177 |  |
| A17_maxbin.001 | 59.55 | 0.97 | 0.00 | 0.86 | 52.6 | Archaea | Thermoproteota | Nitrososphaeria | Nitrososphaerales | Nitrososphaeraceae | Nitrososphaera |  |
| A17_maxbin.011 | 53.09 | 4.76 | 10.00 | 2.69 | 57.5 | Bacteria | Acidobacteriota | Acidobacteriae | Acidobacteriales | Koribacteraceae |  |  |
| A18_maxbin.007 | 96.76 | 5.88 | 45.45 | 3.35 | 51.3 | Archaea | Thermoproteota | Nitrososphaeria | Nitrososphaerales | Nitrososphaeraceae | Nitrososphaera |  |
| A19_maxbin.004 | 99.03 | 7.77 | 16.67 | 3.17 | 28.8 | Archaea | Thermoproteota | Nitrososphaeria | Nitrososphaerales | Nitrososphaeraceae | TH1177 |  |
| A20_maxbin.002 | 99.03 | 3.88 | 0.00 | 2.81 | 28.6 | Archaea | Thermoproteota | Nitrososphaeria | Nitrososphaerales | Nitrososphaeraceae | TH1177 |  |
| A20_maxbin.057 | 54.62 | 3.45 | 0.00 | 3.71 | 75.0 | Bacteria | Actinobacteriota | Actinomycetia |  |  |  |  |
| A20_maxbin.001 | 51.80 | 2.59 | 20.00 | 0.82 | 53.8 | Archaea | Thermoproteota | Nitrososphaeria | Nitrososphaerales | Nitrososphaeraceae | Nitrososphaera |  |
| A21_maxbin.003 | 99.03 | 5.83 | 14.29 | 2.94 | 28.6 | Archaea | Thermoproteota | Nitrososphaeria | Nitrososphaerales | Nitrososphaeraceae | TH1177 |  |
| A21_maxbin.064 | 53.45 | 2.59 | 50.00 | 4.19 | 74.9 | Bacteria | Actinobacteriota | Actinomycetia |  |  |  |  |
| A21_maxbin.001 | 51.15 | 2.34 | 50.00 | 0.80 | 53.4 | Archaea | Thermoproteota | Nitrososphaeria | Nitrososphaerales | Nitrososphaeraceae | Nitrososphaera |  |
| A22_maxbin.001 | 84.79 | 3.88 | 0.00 | 1.57 | 30.2 | Archaea | Thermoproteota | Nitrososphaeria | Nitrososphaerales | Nitrososphaeraceae | TH1177 |  |
| A23_maxbin.009 | 99.03 | 2.91 | 0.00 | 3.26 | 28.9 | Archaea | Thermoproteota | Nitrososphaeria | Nitrososphaerales | Nitrososphaeraceae | TH1177 |  |
| A23_maxbin.010 | 53.41 | 8.87 | 21.88 | 1.69 | 57.5 | Bacteria | Nitrospirota | Nitrospiria | Nitrospirales | Nitrospiraceae | Nitrospira |  |
| A24_maxbin.013 | 99.03 | 5.83 | 0.00 | 3.15 | 28.8 | Archaea | Thermoproteota | Nitrososphaeria | Nitrososphaerales | Nitrososphaeraceae | TH1177 |  |
| A24_maxbin.002 | 88.04 | 2.99 | 25.00 | 1.86 | 52.2 | Archaea | Thermoproteota | Nitrososphaeria | Nitrososphaerales | Nitrososphaeraceae | Nitrososphaera |  |
| A27_maxbin.002 | 79.94 | 2.91 | 0.00 | 1.38 | 30.3 | Archaea | Thermoproteota | Nitrososphaeria | Nitrososphaerales | Nitrososphaeraceae | TH1177 |  |
| A27_maxbin.003 | 70.02 | 6.14 | 42.86 | 1.76 | 40.0 | Bacteria | Firmicutes | Bacilli | Bacillales | Bacillaceae | Priestia | megaterium |
| A27_maxbin.074 | 62.92 | 6.84 | 28.57 | 4.45 | 74.9 | Bacteria | Actinobacteriota | Actinomycetia |  |  |  |  |
| A27_maxbin.001 | 54.56 | 5.95 | 35.71 | 1.05 | 50.9 | Archaea | Thermoproteota | Nitrososphaeria | Nitrososphaerales | Nitrososphaeraceae | Nitrososphaera |  |
| A28_maxbin.013 | 99.03 | 5.34 | 33.33 | 3.24 | 28.9 | Archaea | Thermoproteota | Nitrososphaeria | Nitrososphaerales | Nitrososphaeraceae | TH1177 |  |
| A28_maxbin.005 | 63.30 | 9.26 | 0.00 | 2.72 | 61.2 | Bacteria | Acidobacteriota | Vicinamibacteria | Vicinamibacterales |  |  |  |
| A29_maxbin.006 | 99.03 | 4.85 | 0.00 | 3.11 | 28.7 | Archaea | Thermoproteota | Nitrososphaeria | Nitrososphaerales | Nitrososphaeraceae | TH1177 |  |
| A29_maxbin.001 | 94.66 | 4.99 | 45.45 | 1.74 | 51.6 | Archaea | Thermoproteota | Nitrososphaeria | Nitrososphaerales | Nitrososphaeraceae | Nitrososphaera |  |
| A29_maxbin.002 | 63.93 | 6.25 | 33.33 | 3.18 | 53.4 | Bacteria | Desulfobacterota | Binatia | UBA9968 | UBA9968 | WHTF01 |  |
| A29_maxbin.011 | 63.14 | 6.36 | 0.00 | 3.21 | 49.7 | Bacteria | Chloroflexota | Anaerolineae | Anaerolineales | EnvOPS12 | UBA12294 |  |
| A30_maxbin.002 | 99.03 | 3.88 | 0.00 | 3.07 | 28.6 | Archaea | Thermoproteota | Nitrososphaeria | Nitrososphaerales | Nitrososphaeraceae | TH1177 |  |
| A31_maxbin.002 | 99.03 | 4.85 | 0.00 | 3.10 | 28.6 | Archaea | Thermoproteota | Nitrososphaeria | Nitrososphaerales | Nitrososphaeraceae | TH1177 |  |
| A32_maxbin.008 | 57.51 | 2.43 | 25.00 | 1.53 | 35.7 | Archaea | Thermoproteota | Nitrososphaeria | Nitrososphaerales | Nitrososphaeraceae | TH5893 |  |
| A33_maxbin.006 | 100.00 | 6.80 | 11.11 | 3.01 | 28.6 | Archaea | Thermoproteota | Nitrososphaeria | Nitrososphaerales | Nitrososphaeraceae | TH1177 |  |
| A34_maxbin.006 | 99.03 | 3.88 | 0.00 | 2.88 | 28.6 | Archaea | Thermoproteota | Nitrososphaeria | Nitrososphaerales | Nitrososphaeraceae | TH1177 |  |
| A34_maxbin.001 | 66.67 | 3.24 | 40.00 | 0.97 | 52.7 | Archaea | Thermoproteota | Nitrososphaeria | Nitrososphaerales | Nitrososphaeraceae | Nitrososphaera |  |
| A34_maxbin.058 | 63.40 | 8.62 | 50.00 | 4.69 | 74.8 | Bacteria | Actinobacteriota | Actinomycetia |  |  |  |  |
| A35_maxbin.005 | 93.20 | 3.88 | 0.00 | 2.81 | 28.5 | Archaea | Thermoproteota | Nitrososphaeria | Nitrososphaerales | Nitrososphaeraceae | TH1177 |  |
| A35_maxbin.002 | 63.63 | 6.77 | 10.34 | 3.03 | 53.3 | Bacteria | Desulfobacterota | Binatia | UBA9968 | UBA9968 | WHTF01 |  |
| A35_maxbin.001 | 58.03 | 3.24 | 25.00 | 0.90 | 53.2 | Archaea | Thermoproteota | Nitrososphaeria | Nitrososphaerales | Nitrososphaeraceae | Nitrososphaera |  |
| A36_maxbin.003 | 94.82 | 2.91 | 0.00 | 2.87 | 28.7 | Archaea | Thermoproteota | Nitrososphaeria | Nitrososphaerales | Nitrososphaeraceae | TH1177 |  |
| A36_maxbin.004 | 67.70 | 6.04 | 17.39 | 2.55 | 38.5 | Bacteria | Firmicutes | Bacilli | Bacillales | Bacillaceae | Priestia | megaterium |
| A36_maxbin.024 | 51.42 | 9.02 | 10.91 | 2.53 | 62.5 | Bacteria | Proteobacteria | Gammaproteobacteria | Steroidobacterales | Steroidobacteraceae | PALSA-1196 |  |
| A36_maxbin.059 | 50.86 | 6.03 | 0.00 | 4.13 | 75.0 | Bacteria | Actinobacteriota | Actinomycetia |  |  |  |  |
| A37_maxbin.017 | 99.03 | 9.71 | 0.00 | 3.73 | 28.9 | Archaea | Thermoproteota | Nitrososphaeria | Nitrososphaerales | Nitrososphaeraceae | TH1177 |  |
| A37_maxbin.002 | 92.31 | 8.44 | 25.00 | 3.85 | 68.8 | Bacteria | Gemmatimonadota | Gemmatimonadetes | Gemmatimonadales | GWC2-71-9 | JABFSM01 |  |
| A37_maxbin.018 | 67.56 | 3.88 | 0.00 | 1.53 | 34.9 | Archaea | Thermoproteota | Nitrososphaeria | Nitrososphaerales | Nitrososphaeraceae | TA-21 | sp014523495 |
| A37_maxbin.047 | 59.02 | 5.50 | 57.14 | 2.35 | 68.7 | Bacteria | Chloroflexota | Limnocylindria | Limnocylindrales | CSP1-4 |  |  |
| A38_maxbin.005 | 77.35 | 3.88 | 0.00 | 2.52 | 28.3 | Archaea | Thermoproteota | Nitrososphaeria | Nitrososphaerales | Nitrososphaeraceae | TH1177 |  |
| A39_maxbin.004 | 92.23 | 2.91 | 0.00 | 2.79 | 28.5 | Archaea | Thermoproteota | Nitrososphaeria | Nitrososphaerales | Nitrososphaeraceae | TH1177 |  |
| A39_maxbin.002 | 58.46 | 7.43 | 15.79 | 2.85 | 53.2 | Bacteria | Desulfobacterota | Binatia | UBA9968 | UBA9968 | WHTF01 |  |
| A39_maxbin.001 | 51.78 | 1.94 | 0.00 | 0.85 | 53.2 | Archaea | Thermoproteota | Nitrososphaeria | Nitrososphaerales | Nitrososphaeraceae | Nitrososphaera |  |
| A40_maxbin.001 | 89.97 | 8.33 | 10.00 | 1.73 | 51.9 | Archaea | Thermoproteota | Nitrososphaeria | Nitrososphaerales | Nitrososphaeraceae | Nitrososphaera |  |
| A41_maxbin.022 | 98.54 | 6.80 | 0.00 | 3.64 | 28.9 | Archaea | Thermoproteota | Nitrososphaeria | Nitrososphaerales | Nitrososphaeraceae | TH1177 |  |
| A41_maxbin.010 | 95.98 | 8.10 | 1.47 | 4.31 | 67.0 | Bacteria | Proteobacteria | Gammaproteobacteria | Xanthomonadales | Xanthomonadaceae | Stenotrophomonas | maltophilia |
| A41_maxbin.042 | 65.95 | 7.95 | 60.00 | 2.68 | 68.5 | Bacteria | Chloroflexota | Limnocylindria | Limnocylindrales | CSP1-4 |  |  |
| A41_maxbin.016 | 65.25 | 6.69 | 21.28 | 3.35 | 63.5 | Bacteria | Actinobacteriota | Actinomycetia | Mycobacteriales | Mycobacteriaceae | Rhodococcus | erythropolis |
| A41_maxbin.018 | 64.24 | 6.47 | 18.60 | 3.37 | 43.2 | Bacteria | Firmicutes | Bacilli | Bacillales | DSM-1321 | Peribacillus |  |
| A41_maxbin.040 | 52.59 | 5.17 | 33.33 | 2.43 | 70.7 | Bacteria | Chloroflexota | Limnocylindria | Limnocylindrales | CSP1-4 | SPCO01 |  |
| A42_maxbin.003 | 100.00 | 8.25 | 0.00 | 3.47 | 28.9 | Archaea | Thermoproteota | Nitrososphaeria | Nitrososphaerales | Nitrososphaeraceae | TH1177 |  |
| A42_maxbin.006 | 77.98 | 4.22 | 26.19 | 3.29 | 38.5 | Bacteria | Firmicutes | Bacilli | Bacillales | Bacillaceae | Priestia | megaterium |
| A43_maxbin.010 | 99.03 | 2.91 | 0.00 | 3.43 | 28.9 | Archaea | Thermoproteota | Nitrososphaeria | Nitrososphaerales | Nitrososphaeraceae | TH1177 |  |
| A43_maxbin.002 | 66.69 | 5.49 | 72.73 | 1.29 | 50.1 | Archaea | Thermoproteota | Nitrososphaeria | Nitrososphaerales | Nitrososphaeraceae | Nitrososphaera |  |
| A45_maxbin.007 | 99.03 | 5.83 | 11.11 | 3.28 | 28.6 | Archaea | Thermoproteota | Nitrososphaeria | Nitrososphaerales | Nitrososphaeraceae | TH1177 |  |
| A45_maxbin.009 | 96.41 | 3.92 | 28.57 | 4.65 | 38.4 | Bacteria | Firmicutes | Bacilli | Bacillales | Bacillaceae | Priestia | megaterium |
| A45_maxbin.001 | 65.53 | 0.97 | 0.00 | 1.01 | 52.3 | Archaea | Thermoproteota | Nitrososphaeria | Nitrososphaerales | Nitrososphaeraceae | Nitrososphaera |  |
| A45_maxbin.072 | 57.76 | 7.76 | 40.00 | 5.00 | 74.6 | Bacteria | Actinobacteriota | Actinomycetia |  |  |  |  |
| A45_maxbin.025 | 55.97 | 6.92 | 22.22 | 2.04 | 58.5 | Bacteria | Acidobacteriota | Blastocatellia | Pyrinomonadales | Pyrinomonadaceae | OLB17 |  |
| A46_maxbin.003 | 99.03 | 5.83 | 0.00 | 2.99 | 28.5 | Archaea | Thermoproteota | Nitrososphaeria | Nitrososphaerales | Nitrososphaeraceae | TH1177 |  |
| A47_maxbin.005 | 94.17 | 3.88 | 0.00 | 2.94 | 28.6 | Archaea | Thermoproteota | Nitrososphaeria | Nitrososphaerales | Nitrososphaeraceae | TH1177 |  |
| A47_maxbin.008 | 78.79 | 6.91 | 46.15 | 1.91 | 39.8 | Bacteria | Firmicutes | Bacilli | Bacillales | Bacillaceae | Priestia | megaterium |
| A47_maxbin.001 | 64.08 | 3.24 | 25.00 | 0.94 | 52.6 | Archaea | Thermoproteota | Nitrososphaeria | Nitrososphaerales | Nitrososphaeraceae | Nitrososphaera |  |
| A48_maxbin.006 | 86.23 | 3.88 | 0.00 | 2.79 | 28.5 | Archaea | Thermoproteota | Nitrososphaeria | Nitrososphaerales | Nitrososphaeraceae | TH1177 |  |
| A48_maxbin.001 | 75.51 | 1.94 | 0.00 | 1.30 | 52.4 | Archaea | Thermoproteota | Nitrososphaeria | Nitrososphaerales | Nitrososphaeraceae | Nitrososphaera |  |
| A48_maxbin.066 | 59.83 | 7.12 | 0.00 | 4.69 | 74.4 | Bacteria | Actinobacteriota |  |  |  |  |  |
| A49_maxbin.001 | 92.72 | 1.94 | 0.00 | 1.80 | 51.3 | Archaea | Thermoproteota | Nitrososphaeria | Nitrososphaerales | Nitrososphaeraceae | Nitrososphaera |  |
| A49_maxbin.064 | 64.66 | 8.62 | 25.00 | 4.76 | 74.8 | Bacteria | Actinobacteriota | Actinomycetia |  |  |  |  |
| A49_maxbin.008 | 60.45 | 5.08 | 12.50 | 2.62 | 68.2 | Bacteria | Actinobacteriota | Acidimicrobiia | IMCC26256 | PALSA-610 |  |  |
| A49_maxbin.002 | 56.18 | 0.94 | 33.33 | 1.97 | 38.8 | Bacteria | Firmicutes | Bacilli | Bacillales | Bacillaceae | Priestia | megaterium |
| A49_maxbin.015 | 50.05 | 2.81 | 21.43 | 1.76 | 55.8 | Bacteria | Proteobacteria | Gammaproteobacteria | JACCYU01 | JACCYU01 |  |  |
| A50_maxbin.010 | 99.03 | 5.83 | 0.00 | 3.15 | 28.5 | Archaea | Thermoproteota | Nitrososphaeria | Nitrososphaerales | Nitrososphaeraceae | TH1177 |  |
| A50_maxbin.001 | 76.91 | 0.49 | 0.00 | 1.31 | 52.4 | Archaea | Thermoproteota | Nitrososphaeria | Nitrososphaerales | Nitrososphaeraceae | Nitrososphaera |  |
| A51_maxbin.004 | 99.03 | 4.85 | 0.00 | 2.91 | 28.6 | Archaea | Thermoproteota | Nitrososphaeria | Nitrososphaerales | Nitrososphaeraceae | TH1177 |  |
| A52_maxbin.114 | 56.36 | 5.50 | 21.43 | 2.57 | 63.9 | Bacteria | Proteobacteria | Alphaproteobacteria | Rhizobiales | Beijerinckiaceae | Microvirga |  |
| A53_maxbin.010 | 99.03 | 6.87 | 12.50 | 3.35 | 28.9 | Archaea | Thermoproteota | Nitrososphaeria | Nitrososphaerales | Nitrososphaeraceae | TH1177 |  |
| A53_maxbin.006 | 54.41 | 3.08 | 28.57 | 1.89 | 38.6 | Bacteria | Firmicutes | Bacilli | Bacillales | Bacillaceae | Priestia | megaterium |
| A53_maxbin.008 | 50.62 | 8.12 | 0.00 | 2.49 | 68.2 | Bacteria | Actinobacteriota | Acidimicrobiia | IMCC26256 | PALSA-610 |  |  |
| A54_maxbin.003 | 50.06 | 4.39 | 19.05 | 1.61 | 68.4 | Bacteria | Actinobacteriota | UBA4738 | UBA4738 | UBA4738 |  |  |
| A55_maxbin.002 | 99.03 | 5.34 | 14.29 | 3.28 | 28.8 | Archaea | Thermoproteota | Nitrososphaeria | Nitrososphaerales | Nitrososphaeraceae | TH1177 |  |
| A55_maxbin.024 | 58.06 | 7.41 | 30.77 | 3.01 | 68.5 | Bacteria | Chloroflexota | Limnocylindria | Limnocylindrales | CSP1-4 |  |  |
| A55_maxbin.007 | 50.48 | 3.86 | 26.32 | 2.57 | 57.0 | Bacteria | Acidobacteriota | Acidobacteriae | Acidobacteriales | Koribacteraceae |  |  |
| A56_maxbin.002 | 93.20 | 2.91 | 0.00 | 2.90 | 28.5 | Archaea | Thermoproteota | Nitrososphaeria | Nitrososphaerales | Nitrososphaeraceae | TH1177 |  |
| A56_maxbin.020 | 74.55 | 8.28 | 11.11 | 2.37 | 57.9 | Bacteria | Nitrospirota | Nitrospiria | Nitrospirales | Nitrospiraceae | Nitrospira |  |
| A56_maxbin.006 | 56.02 | 7.87 | 29.41 | 2.94 | 69.0 | Bacteria | Gemmatimonadota | Gemmatimonadetes | Gemmatimonadales | GWC2-71-9 | JABFSM01 |  |
| A57_maxbin.007 | 71.19 | 8.45 | 33.33 | 3.66 | 57.2 | Bacteria | Acidobacteriota | Acidobacteriae | Acidobacteriales | Koribacteraceae |  |  |
| A57_maxbin.091 | 68.22 | 9.22 | 80.00 | 1.22 | 56.4 | Archaea | Thermoproteota | Nitrososphaeria | Nitrososphaerales | Nitrososphaeraceae | Nitrososphaera |  |
| A57_maxbin.013 | 61.76 | 5.43 | 14.63 | 2.13 | 55.8 | Bacteria | Proteobacteria | Gammaproteobacteria | JACCYU01 | JACCYU01 |  |  |
| A57_maxbin.014 | 57.12 | 5.83 | 42.86 | 1.07 | 52.2 | Archaea | Thermoproteota | Nitrososphaeria | Nitrososphaerales | Nitrososphaeraceae | Nitrososphaera |  |
| A57_maxbin.056 | 54.76 | 8.97 | 17.39 | 3.13 | 65.4 | Bacteria | Chloroflexota | Chloroflexia | 54-19 | JADMIH01 |  |  |
| A58_maxbin.010 | 100.00 | 8.25 | 0.00 | 3.20 | 28.7 | Archaea | Thermoproteota | Nitrososphaeria | Nitrososphaerales | Nitrososphaeraceae | TH1177 |  |
| A58_maxbin.004 | 63.80 | 8.83 | 9.09 | 2.84 | 68.3 | Bacteria | Actinobacteriota | Acidimicrobiia | IMCC26256 | PALSA-610 |  |  |
| A59_maxbin.008 | 99.03 | 6.80 | 12.50 | 3.05 | 28.6 | Archaea | Thermoproteota | Nitrososphaeria | Nitrososphaerales | Nitrososphaeraceae | TH1177 |  |
| A59_maxbin.001 | 93.04 | 4.85 | 16.67 | 1.76 | 51.7 | Archaea | Thermoproteota | Nitrososphaeria | Nitrososphaerales | Nitrososphaeraceae | Nitrososphaera |  |
| A59_maxbin.002 | 52.13 | 2.16 | 28.57 | 1.67 | 39.9 | Bacteria | Firmicutes | Bacilli | Bacillales | Bacillaceae | Priestia | megaterium |
| A60_maxbin.003 | 88.35 | 4.85 | 0.00 | 2.84 | 28.5 | Archaea | Thermoproteota | Nitrososphaeria | Nitrososphaerales | Nitrososphaeraceae | TH1177 |  |
| A60_maxbin.001 | 53.48 | 3.88 | 25.00 | 0.88 | 53.4 | Archaea | Thermoproteota | Nitrososphaeria | Nitrososphaerales | Nitrososphaeraceae | Nitrososphaera |  |
| A61_maxbin.005 | 99.03 | 6.80 | 0.00 | 3.33 | 28.8 | Archaea | Thermoproteota | Nitrososphaeria | Nitrososphaerales | Nitrososphaeraceae | TH1177 |  |
| A61_maxbin.001 | 94.82 | 8.78 | 67.50 | 1.87 | 51.4 | Archaea | Thermoproteota | Nitrososphaeria | Nitrososphaerales | Nitrososphaeraceae | Nitrososphaera |  |
| A61_maxbin.004 | 81.37 | 6.49 | 38.10 | 3.26 | 38.5 | Bacteria | Firmicutes | Bacilli | Bacillales | Bacillaceae | Priestia | megaterium |
| A61_maxbin.002 | 61.54 | 7.93 | 2.86 | 3.03 | 68.2 | Bacteria | Actinobacteriota | Acidimicrobiia | IMCC26256 | PALSA-610 |  |  |
| A61_maxbin.010 | 50.11 | 6.55 | 13.04 | 2.96 | 57.0 | Bacteria | Acidobacteriota | Acidobacteriae | Acidobacteriales | Koribacteraceae |  |  |
| A62_maxbin.012 | 100.00 | 7.28 | 11.11 | 3.60 | 28.9 | Archaea | Thermoproteota | Nitrososphaeria | Nitrososphaerales | Nitrososphaeraceae | TH1177 |  |
| A62_maxbin.009 | 64.18 | 5.67 | 18.75 | 3.43 | 57.0 | Bacteria | Acidobacteriota | Acidobacteriae | Acidobacteriales | Koribacteraceae |  |  |
| A62_maxbin.045 | 50.68 | 4.57 | 0.00 | 2.91 | 65.5 | Bacteria | Chloroflexota | Chloroflexia | 54-19 | JADMIH01 |  |  |
| A63_maxbin.004 | 99.03 | 4.85 | 0.00 | 2.98 | 28.7 | Archaea | Thermoproteota | Nitrososphaeria | Nitrososphaerales | Nitrososphaeraceae | TH1177 |  |
| A63_maxbin.001 | 88.03 | 6.91 | 43.75 | 1.63 | 51.8 | Archaea | Thermoproteota | Nitrososphaeria | Nitrososphaerales | Nitrososphaeraceae | Nitrososphaera |  |
| A63_maxbin.059 | 54.31 | 3.45 | 0.00 | 4.74 | 74.8 | Bacteria | Actinobacteriota |  |  |  |  |  |
|  |  |  |  |  |  |  |  |  |  |  |  |  |
|  |  |  |  |  |  |  |  |  |  |  |  |  |
|  |  |  |  |  |  |  |  |  |  |  |  |  |
|  |  |  |  |  |  |  |  |  |  |  |  |  |
|  |  |  |  |  |  |  |  |  |  |  |  |  |
