# Supplementary material for: Multi-omics resolved integration reveals microbial niche separation in soil aggregates
Source: ISME Commun. 2026 Jun 11;6(1):ycag161. doi: 10.1093/ismeco/ycag161 (PMC13398701; doi:10.1093/ismeco/ycag161)

Multi-omics resolved integration reveals microbial niche separation in soil aggregates

SUPPLEMENTARY MATERIALS

Jonathan Y. Lin[1], Júlia Brandão Gontijo[1], Cameron K. McMillan[1], Jane D. Fudyma[2], Daoyuan Wang[3], Erika H. Yao[1], Jordan M. Sayre[1], Joanne B. Emerson[2], David A. Lipson[4], Cristina Lazcano[1], Kate M. Scow[1], Jorge L. Mazza Rodrigues[1,5*]

[1]Department of Land, Air, and Water Resources, University of California, Davis, CA, USA 95616
[2]Department of Plant Pathology, University of California, Davis, CA, USA 95616
[3]Department of Environmental Science and Engineering, Shanghai University, Shanghai, People's Republic of China 200444
[4]Department of Biology, San Diego State University, San Diego, CA, USA 92182
[5]Environmental Genomics and Systems Biology Division, Lawrence Berkeley National Laboratory, Berkeley, CA, USA 94720

*Correspondence: jmrodrigues@ucdavis.edu

SUPPLEMENTARY METHODS

Diversity Analysis

Richness, Shannon diversity and Pielou's evenness were calculated from taxonomic abundance tables generated from Kraken2 [1] algorithm. Alpha- and beta-diversity analyses based on Kraken2 profiles were performed using species-level taxonomic abundance tables. Additionally, marker gene-based prokaryotic community profiling was performed using the SingleM v0.21.0 [2] algorithm with the GTDB-based metapackage S5.4.0.GTDB_r226, based on Genome Taxonomy Database release R226. SingleM was run independently for each metagenomic sample, generating sample-level marker-gene OTU tables based on conserved windows within 59 universal single-copy marker genes (**Supplementary Table ST2**). These OTU tables were subsequently merged into a combined sample-by-OTU matrix for downstream diversity analyses. OTUs were taxonomically classified down to the species level whenever possible. Alpha- and beta-diversity metrics were calculated at the OTU level. Metrics were computed in R using the *vegan* package [3].

Quantitative PCR

Owing to the significant differences in metagenome-assembled genome abundances of ammonia-oxidizing archaea in soil aggregates, we used quantitative PCR (qPCR) to verify our bioinformatic results. The primers crenamo A23f/crenamo A616r (4), which target the *amoA* gene responsible for encoding the ammonia monooxygenase enzyme in archaea, were used. Amplifications were performed on a Bio-Rad CFX Connect System (Bio-Rad Laboratories, Hercules, CA, USA) in 20 µL reaction mixtures containing 10 µL SsoAdvanced Universal SYBR Green Supermix (Bio-Rad Laboratories, Hercules, CA, USA), 0.5 µM each primer, 5 ng template DNA, and 4 µL sterile ddH_2O. Standard curves with a detection range of 10^2-10^8 copies were generated with the pCR Blunt II-TOPO vector (Invitrogen Co., Waltham, MA, USA) containing

PCR-amplified fragments from environmental samples. Thermal cycling conditions were initiated with a 5 min at 95°C , followed by 10 cycles of 94°C for 30 s, annealing at 55°C for 30 s, and extension at 72°C for 1min; followed by 25 cycles of 92°C for 30 s, 55°C for 30 s, 72°C for 1min; followed by 72°C at 10 min, according to protocol described in Tourna *et al.* (59). Triplicate reactions were performed for each sample and a melting curve analysis was run to ensure specificity of the amplified products. The coefficient of determination (R^2) and amplification efficiency was 0.927 and 99.93%, respectively.

Untargeted metabolomics

A total of eight samples for each one of the four aggregate size fractions, totaling were 32 samples, were assayed for untargeted metabolomics to test whether differences in functional gene potential in aggregates translated into metabolic output differences. Aliquots for each aggregate size fraction were either immediately frozen at -80°C until submission to the UC Davis West Coast Metabolomics Center for untargeted analysis of primary metabolites using a LECO Pegasus GC-TOF MS (St. Joseph, MI, USA) (5). The metabolites analyzed included carbohydrates and sugar phosphates, amino acids, hydroxyl acids, free fatty acids, purines, pyrimidines, aromatics, and exposome-derived chemicals.

Enzymatic assays

The same 32 samples collected for metabolomics were also assayed for seven different enzymes. Owing to the agricultural nature of the study, we selected microbial enzymes involved in the cycling of carbon (C), nitrogen (N) and phosphorus (P). Four enzymes were C-cycling enzymes, namely: β-D-cellobiosidase, β-xylosidase, α-glucosidase, β-glucosidase; two enzymes are related to nitrogen availability through chitin degradation (N-acetyl-β-glucosaminidase) and proteolytic activity (leucine aminopeptidase); and one P-cycling enzyme, responsible for hydrolysis of organic P compounds (phosphatase). Soil extracellular enzymes were assayed

according to Bell *et. al* (6). Briefly, 2.75 g of soil from each aggregate size fraction was blended in 50 mM tris buffer (pH 7.2) to create a slurry, incubated with 4-methylumbelliferone (MUB) or 7-amino-4-methylcoumarin (MUC)-linked substrates for 3 h, and read on a microplate fluorimeter (Agilent, Santa Clara, CA, USA).. Enzyme activities are reported per gram of aggregate size fraction.

Supplementary References

1. Wood DE, Lu J, Langmead B. Improved metagenomic analysis with Kraken 2. *Genome Biol* 2019; **20**: 257.
2. Woodcroft BJ, Aroney STN, Zhao R, Cunningham M, Mitchell JAM, Nurdiansyah R, Blackall L, Tyson GW. Comprehensive taxonomic identification of microbial species in metagenomic data using SingleM and Sandpiper. *Nature Biotechnol* 2025; https://doi.org/10.1038/s41587-025-02738-1
3. Oksanen J, Blanchet FG, Friendly M, Kindt R, Legendre P, McGlinn D, et al. vegan: Community ecology package. 2019.
4. Tourna M, Freitag TE, Nicol GW, Prosser JI. Growth, activity and temperature responses of ammonia-oxidizing archaea and bacteria in soil microcosms. *Environ Microbiol* 2008; **10**: 1357–1364.
5. Lai Z, Tsugawa H, Wohlgemuth G, Mehta S, Mueller M, Zheng Y, et al. Identifying metabolites by integrating metabolome databases with mass spectrometry cheminformatics. *Nat Methods* 2018; **15**: 53–56.
6. Bell CW, Fricks BE, Rocca JD, Steinweg JM, McMahon SK, Wallenstein MD. High-throughput fluorometric measurement of potential soil extracellular enzyme activities. *J Vis Exp* 2013; e50961.

SUPPLEMENTARY TABLES

Supplementary Table ST1. Metagenome assembly metrics of all samples used in this study.

Sample	Reads passing filter	Contigs (#)	Total length (bp)	Max. contig size (bp)	Avg. contig Size (bp)	N50
A1	93,068,016	408,657	720,777,762	66,484	1,764	1,726
A2	96,483,071	429,998	761,324,419	92,951	1,771	1,733
A3	88,264,717	371,426	639,173,091	69,751	1,721	1,669
A4	84,358,174	371,595	640,454,282	52,254	1,724	1,673
A5	83,701,364	364,059	619,293,274	52,212	1,701	1,650
A6	98,452,482	450,066	781,859,709	61,727	1,737	1,689
A7	81,309,855	339,679	578,017,829	59,468	1,702	1,646
A8	79,654,292	328,613	561,746,196	63,886	1,709	1,658
A9	82,490,151	342,004	602,316,569	73,303	1,761	1,712
A10	85,728,088	389,354	677,773,968	67,438	1,741	1,695
A11	76,999,523	293,301	497,961,869	65,610	1,698	1,637
A12	56,200,127	198,390	335,033,874	46,902	1,689	1,628
A13	80,699,188	354,840	613,951,980	60,341	1,730	1,679
A14	74,746,139	299,182	508,993,070	52,867	1,701	1,644
A15	92,664,020	368,254	625,650,265	62,283	1,699	1,642
A16	95,258,212	386,748	665,995,077	64,893	1,722	1,676
A17	89,830,850	356,082	610,969,904	50,049	1,716	1,668
A18	85,068,952	353,399	614,153,011	60,623	1,738	1,687
A19	91,955,110	346,781	589,662,339	72,638	1,700	1,645
A20	73,364,464	271,393	466,922,249	63,290	1,720	1,662
A21	73,273,468	301,636	514,483,438	62,127	1,706	1,649
A22	88,927,291	352,477	603,482,913	62,187	1,712	1,661
A23	78,653,266	285,858	484,651,061	66,059	1,695	1,633
A24	75,633,534	280,247	471,487,654	54,938	1,682	1,627
A25	97,469,181	449,521	798,580,696	64,333	1,777	1,742
A26	106,652,794	473,287	832,986,618	65,924	1,760	1,725
A27	83,809,942	333,195	567,738,222	61,651	1,704	1,645
A28	89,565,789	392,346	682,537,564	59,702	1,740	1,694
A29	82,254,539	341,799	584,275,980	65,925	1,709	1,659
A30	86,247,448	348,824	599,827,028	76,909	1,720	1,666
A31	77,747,030	265,642	450,021,323	92,267	1,694	1,632
A32	27,974,166	55,224	87,981,554	24,348	1,593	1,517
A33	75,093,158	244,992	411,408,434	63,396	1,679	1,613
A34	75,249,687	273,284	467,743,067	57,961	1,712	1,662
A35	75,378,768	252,217	424,007,364	59,301	1,681	1,618
A36	78,787,908	305,065	519,856,584	60,999	1,704	1,647
A37	85,835,752	399,565	700,883,231	86,352	1,754	1,713
A38	80,340,447	303,709	539,868,142	49,513	1,778	1,735
A39	54,799,017	184,998	308,331,957	53,885	1667	1,603
A40	71,419,450	291,586	499,900,323	59,178	1,714	1,656
A41	94,497,999	369,615	669,227,326	467,732	1,811	1,749
A42	93,724,606	400,107	691,174,139	74,112	1,727	1,682
A43	101,610,750	427,418	733,380,470	61,348	1,716	1,667
A44	87,602,634	369,022	634,789,340	64,933	1,720	1,671
A45	81,136,375	336,844	572,431,809	42,492	1,699	1,639
A46	79,270,921	315,016	539,344,879	67,001	1,712	1,658
A47	94,551,402	363,028	614,098,008	57,473	1,692	1,631
A48	76,807,798	275,816	463,433,179	61,023	1,680	1,614
A49	98,133,324	441,361	769,726,346	98,747	1,744	1,701
A50	88,492,880	372,559	641,393,435	63,551	1,722	1,685
A51	77,823,368	313,947	537,272,147	67,668	1,711	1,653
A52	65,127,954	267,208	453,898,238	57,822	1,699	1,641
A53	86,116,076	382,423	665,386,970	71,752	1,740	1,693
A54	92,272,665	415,047	723,651,756	53,935	1,744	1,697
A55	90,523,851	384,360	665,591,172	70,479	1,732	1,682
A56	78,539,170	308,870	526,429,786	53,688	1,704	1,644
A57	102,326,661	488,231	856,109,143	69,260	1,753	1,723
A58	96,226,982	447,141	787,761,723	60,487	1,762	1,723
A59	93,460,768	418,521	729,027,907	70,750	1,742	1,696
A60	77,579,732	327,854	562,489,066	61,012	1,716	1,665
A61	82,711,448	384,852	673,624,483	65,432	1,750	1,704
A62	97,496,814	428,067	748,300,399	83,007	1,748	1,710
A63	89,604,825	364,353	626,160,196	63,655	1,719	1,665
A64	96,172,004	397,690	691,187,243	74,390	1,738	1,689

Supplementary Table ST2. List of the 59 marker genes used by SingleM for taxonomic assignment.

S3.1.ribosomal_protein_L2_rplB	S3.31.ribosomal_S24e
S3.2.ribosomal_protein_L3_rplC	S3.32.ribosomal_S28e
S3.3.ribosomal_protein_L14b_L23e_rplN	S3.33.ribosomal_S4e
S3.4.ribosomal_protein_L16_L10E_rplP	S3.34.ribosomal_S6e
S3.5.ribosomal_protein_S2_rpsB	S3.35.RNA_SBDS
S3.6.ribosomal_protein_S5	S3.36.uL22_arch_euk
S3.7.ribosomal_protein_S7	S3.37.uS4_arch
S3.8.ribosomal_protein_S12_S23	S3.38.ribosomal_protein_L5_rplE
S3.9.ribosomal_protein_S15P_S13e	S3.39.ribosomal_protein_L6_rplF
S3.10.ribosomal_protein_S19_rpsS	S3.40.ribosomal_protein_L11_rplK
S3.11.pheS	S3.41.ribosomal_protein_S10_rpsJ
S3.12.ribosomal_L1	S3.42.RNA_pol_A_bac
S3.13.ribosomal_S9	S3.43.TIGR00042
S3.14.hisS	S3.44.alaS
S3.15.glyS_dimeric	S3.45.NusA
S3.16.SRP_SPB	S3.46.pheT_bact
S3.17.dph5	S3.47.recR
S3.18.EIF_2_alpha	S3.48.rplD_bact
S3.19.eIF_5A	S3.49.rplO_bact
S3.20.eS8	S3.50.rplT_bact
S3.21.Fibrillarin	S3.51.rplV_bact
S3.22.gatD_arch	S3.52.rpsC_bact
S3.23.KOW_elon_Spt5	S3.53.ruvA
S3.24.Nop	S3.54.serS
S3.25.ribosomal_L15e	S3.55.TIGR00006
S3.26.ribosomal_L19e	S3.56.trmD
S3.27.ribosomal_L21e	S3.57.TruB
S3.28.ribosomal_L31e	S3.58.tsf
S3.29.ribosomal_L32e	S3.59.uS11_bact
S3.30.ribosomal_S19e	

Supplementary Table ST3. Differential abundance analysis (*P* < 0.05) of microbial taxonomy at the genus level (a), KEGG genes (b), and metabolites (c) by fertilizer management. A positive log_2-fold change value indicates enrichment under mineral fertilizer, while a negative log_2-fold change value indicates enrichment under manure compost.

a)

Taxonomy						
Genus	Log_2FC	Log_2CPM	LR	*P*-value	FDR	Classification
Pseudomonas	-7.05	8.53	100.39	1.25E-23	3.74E-20	Bacteria; Proteobacteria; Gammaproteobacteria; Pseudomonadales; Pseudomonadaceae
Chryseobacterium	-9.15	7.99	141.72	1.12E-32	6.71E-29	Bacteria; Bacteroidetes; Flavobacteriia; Flavobacteriales; Weeksellaceae
Rhodococcus	-1.68	4.62	30.77	2.90E-08	3.47E-06	Bacteria; Actinobacteria; Actinomycetia; Corynebacteriales; Nocardiaceae
Sphingobacterium	-6.58	6.17	91.36	1.20E-21	2.39E-18	Bacteria; Bacteroidetes; Sphingobacteriia; Sphingobacteriales; Sphingobacteriaceae
Pedobacter	-1.11	3.78	12.23	4.69E-04	1.05E-02	Bacteria; Bacteroidetes; Sphingobacteriia; Sphingobacteriales; Sphingobacteriaceae
Rhodanobacter	1.66	7.27	66.96	2.77E-16	2.07E-13	Bacteria; Proteobacteria; Gammaproteobacteria; Xanthomonadales; Rhodanobacteraceae
Pantoea	1.13	4.50	9.89	1.66E-03	2.54E-02	Bacteria; Proteobacteria; Gammaproteobacteria; Enterobacterales; Erwiniaceae
Nitrosospira	1.51	4.80	66.00	4.51E-16	3.00E-13	Bacteria; Proteobacteria; Betaproteobacteria; Nitrososmonadales; Nitrosomonadaceae

b)

Function (KO)						
KEGG ID	Log_2FC	Log_2CPM	LR	*P*-value	FDR	Gene Name
K00496	-0.48	6.17	20.42	6.22E-06	3.79E-03	alkane 1-monooxygenase [EC:1.14.15.3]
K06188	-0.42	6.11	47.85	4.59E-12	1.12E-08	aquaporin Z
K06895	-0.47	5.99	14.83	1.18E-04	1.65E-02	L-exporter protein LysE/ArgO
K18481	-0.46	7.22	18.50	1.70E-05	8.27E-03	Mce-membrane protein
K19577	-0.65	6.13	14.86	1.16E-04	1.65E-02	MFS transporter, family, membrane protein
K21672	-0.49	6.34	36.43	1.58E-09	1.93E-06	2,4-dehydrogenase [EC:1.4.1.1.4.1.26]

c)

Metabolites						
KEGG ID	Log_2FC	Avg. Expression	*t*-statistic	*P*-value	Adj. *P*-value	Compound
C00252	-472.81	467.66	-4.19	0.000	0.013	isomaltose
C00249	20180.38	161777.13	3.58	0.001	0.049	palmitic acid
C01753	-1901.69	1851.28	-3.50	0.001	0.049	beta-sitosterol

Supplementary Table ST4. Differentially abundant KEGG genes ($P < 0.05$) between aggregate size fractions. A positive $\log_2$-fold change value indicates enrichment in the microaggregates and silt & clay, while a negative $\log_2$-fold change value indicates enrichment in the large and small macroaggregates.

(Please, see additional table)

Supplementary Table ST5. Summary metrics and taxonomic classifications of metagenome assembled genomes (MAGs) used in this study.

(Please, see additional table)

SUPPLEMENTARY FIGURES

Supplementary Figure SF1. Alpha diversity of microbial taxonomy (a-c) and function (d-f) based on Richness, Shannon, and Evenness indexes by fertilizer treatment across all samples. Asterisks represent significant differences ($P < 0.05$) between fertilizer treatments, while N.S. represents no significance.

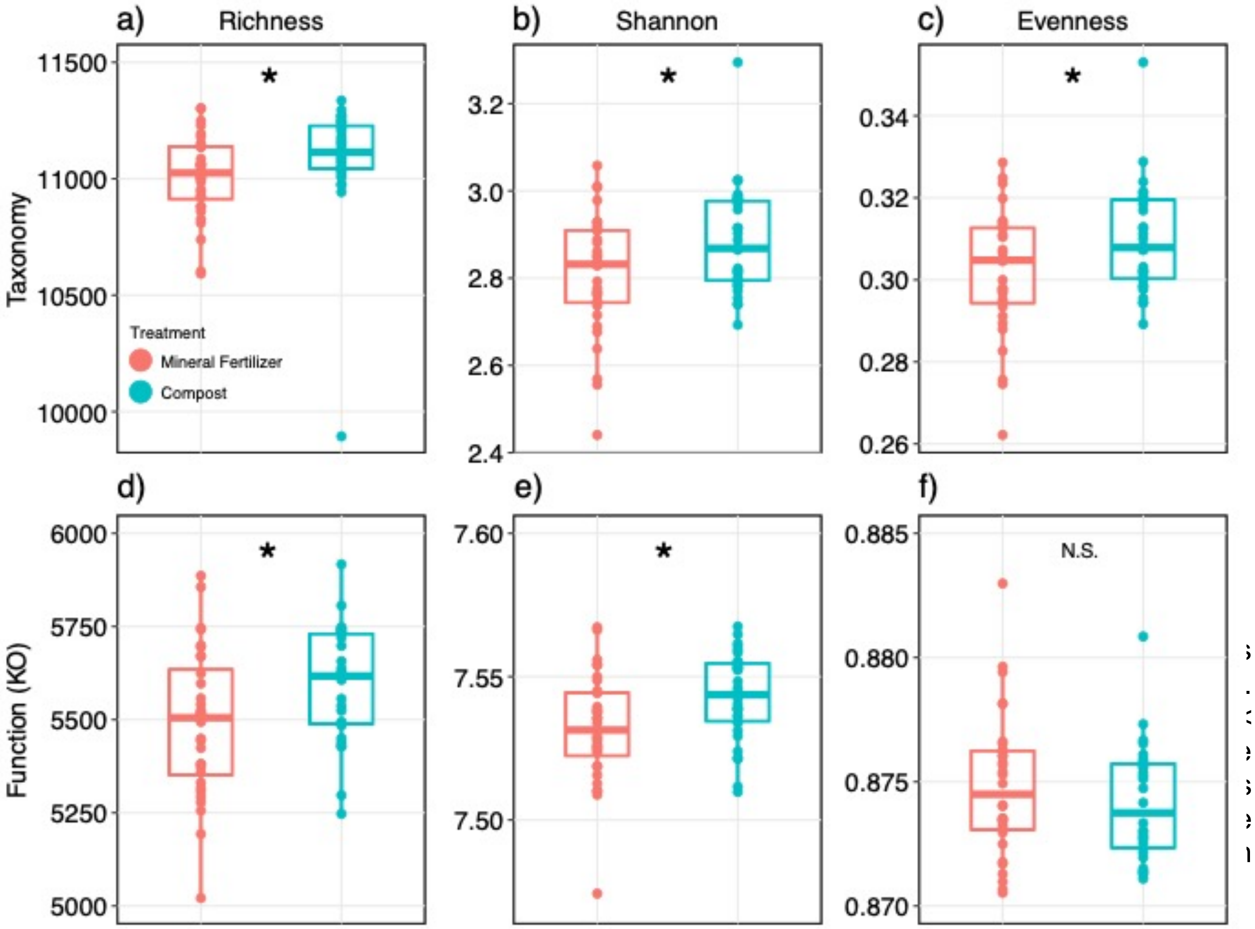

Supplementary Figure SF2. Alpha and beta diversity and prokaryotic community composition across aggregate size classes using SingleM. a) Alpha diversity metrics, including Richness, Shannon diversity, and Pielou's Evenness at OUT level. Different letters indicate statistically significant differences among aggregate size classes ($p < 0.05$). b) NMDS ordination showing differences in prokaryotic community structure among aggregate size classes based on Bray-Curtis dissimilarities calculated at the OUT level. c) Relative abundance of prokaryotic phyla inferred by SingleM GTDB-based taxonomic profiles for each aggregate size class. Values represent mean relative abundance within each aggregate size class. Only phyla with mean relative abundance > 0.05% were retained; all remaining phyla were grouped as "Others".

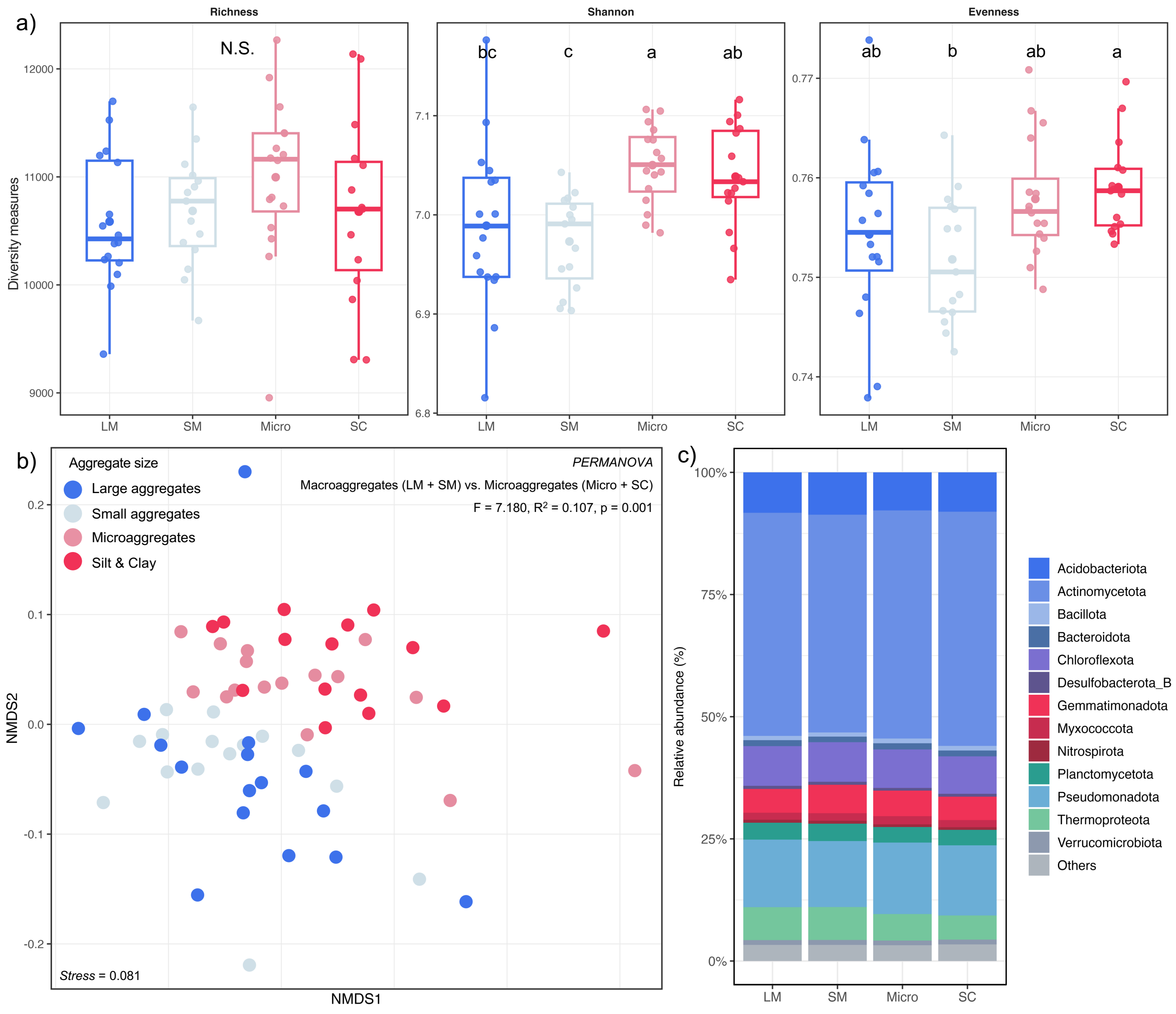

Supplementary Figure. SF3. Pathway analysis showing enriched KEGG pathways based on differentially abundant KO terms. Representative K numbers for each pathway are shown on the x axis. A positive log_2-fold change value indicates enrichment in the microaggregates and silt & clay, while a negative log_2-fold change value indicates enrichment in the large and small macroaggregates.

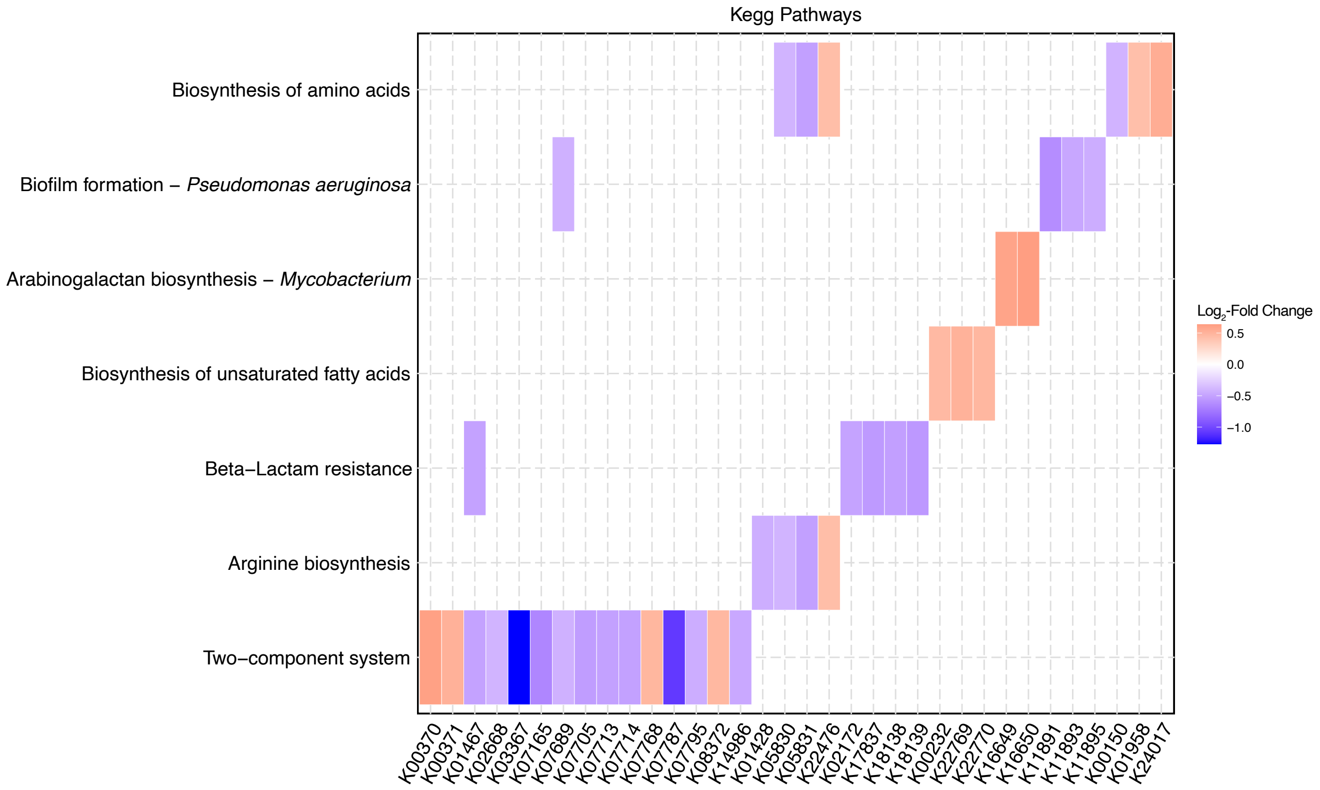

Supplementary Figure SF4. Differentially abundant metabolites by aggregate size. Different letters represent significant differences ($P < 0.05$) between aggregate size fractions.

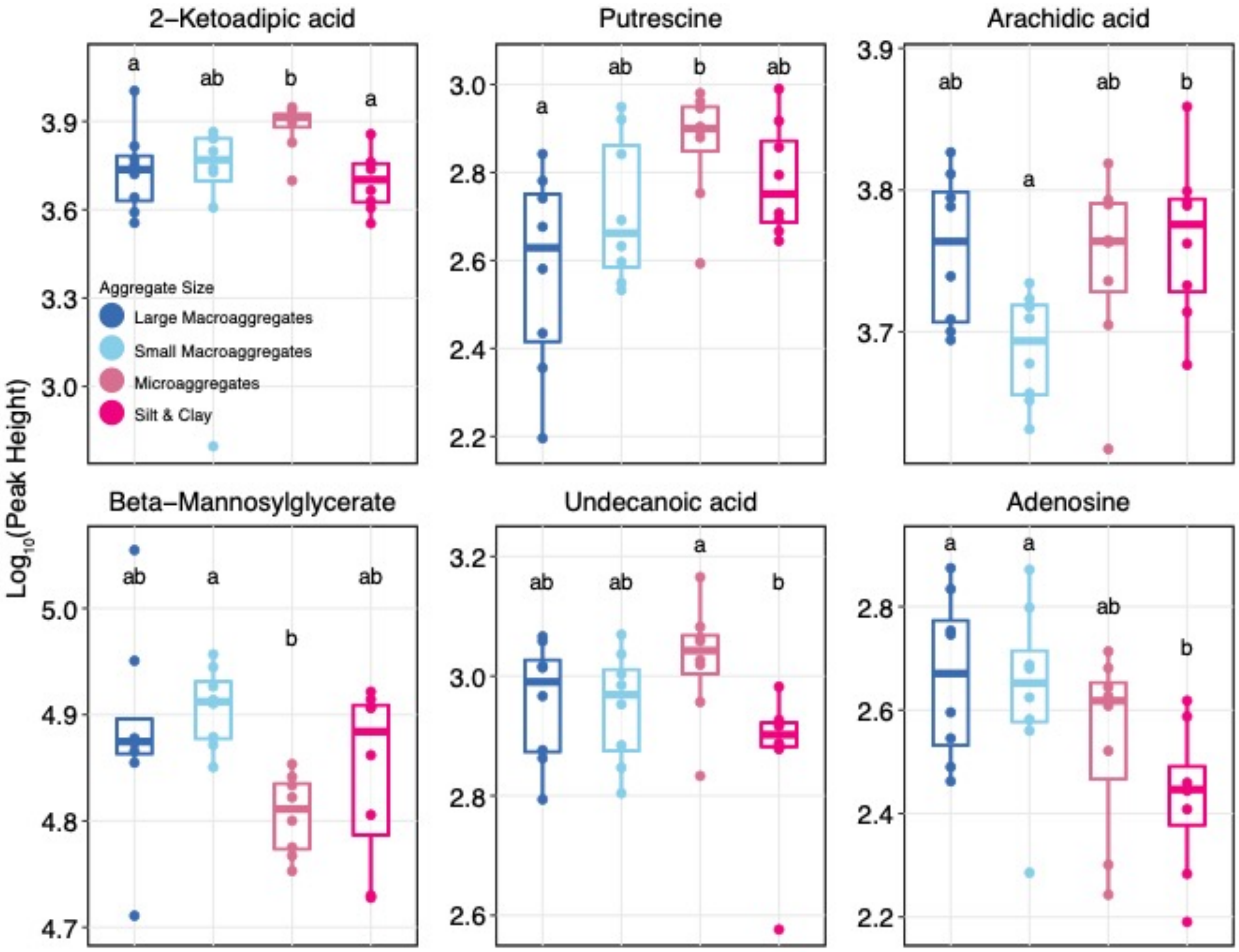

Supplementary Figure SF5. (a) Non-metric multidimensional scaling (NMDS) plot based on the Euclidean distance of enzyme activities of soil aggregates. The ordination stress value was 0.035. (b) Permutational multivariate analysis of variance (PERMANOVA) results for the enzymes and (c) the specific activities for each of the 7 enzymes assayed. Samples in the NMDS are colored by aggregate size and shapes correspond with different fertilizer treatments. Different letters in the boxplots represent significant differences (*P* < 0.05) between aggregate size fractions. Abbreviations for each enzyme are listed in the legend.

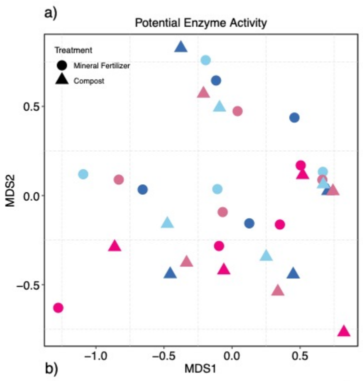

b)

Variable	Df	SumSqs	R^2	F	P-value
Aggregate Size	3	1.117	0.068	0.621	0.740
Treatment	1	0.741	0.045	1.237	0.314
Aggregate Size:Treatment	3	0.110	0.007	0.061	1.000
Residuals	24	14.383	0.880		
Total	31	16.351	1.000		

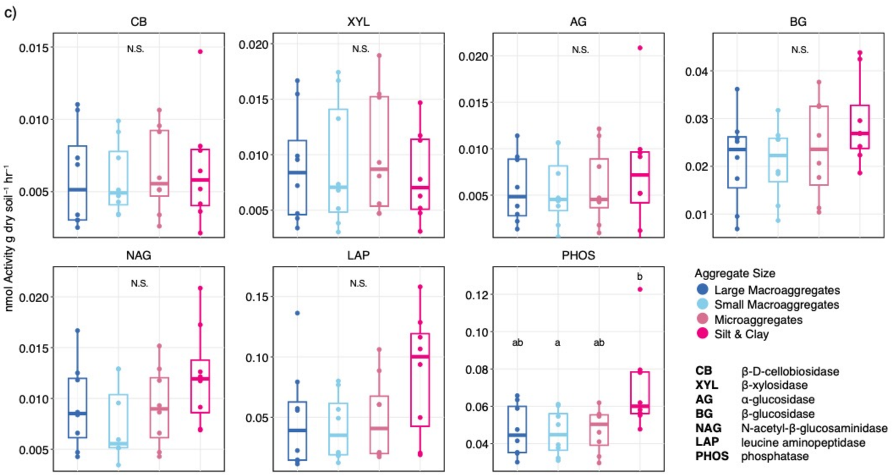

Supplementary Figure SF6. Unrooted phylogenetic trees of bacterial and archaeal (a) metagenome assembled genomes (MAGs) used in this study. The phylogenetic tree was constructed based on concatenated alignments of 49 universal single-copy marker genes. (b) Number of MAGs retrieved for each phylum by aggregate size.

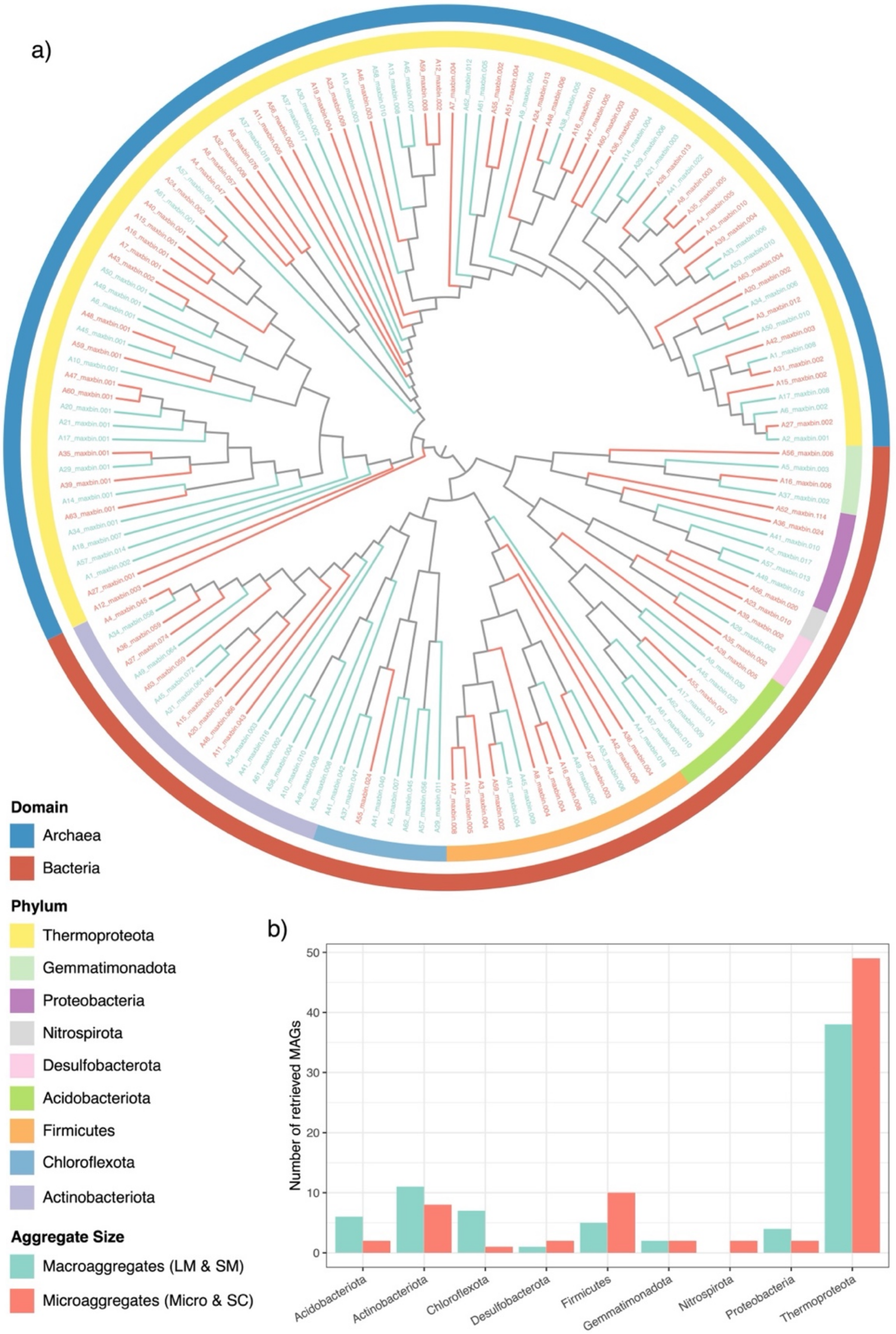

Supplementary Figure SF7. Phylogenetic tree of high-quality archaea MAGs used in this study. The tree, which was constructed using multiple sequence alignments of 53 single-copy marker genes, was trimmed to visualize MAG placement within the order *Nitrososphaerales*. Genome taxonomy database (GTDB) names are given at branch nodes and numbers indicate bootstrap support. Bar, 0.1 substitutions per position.

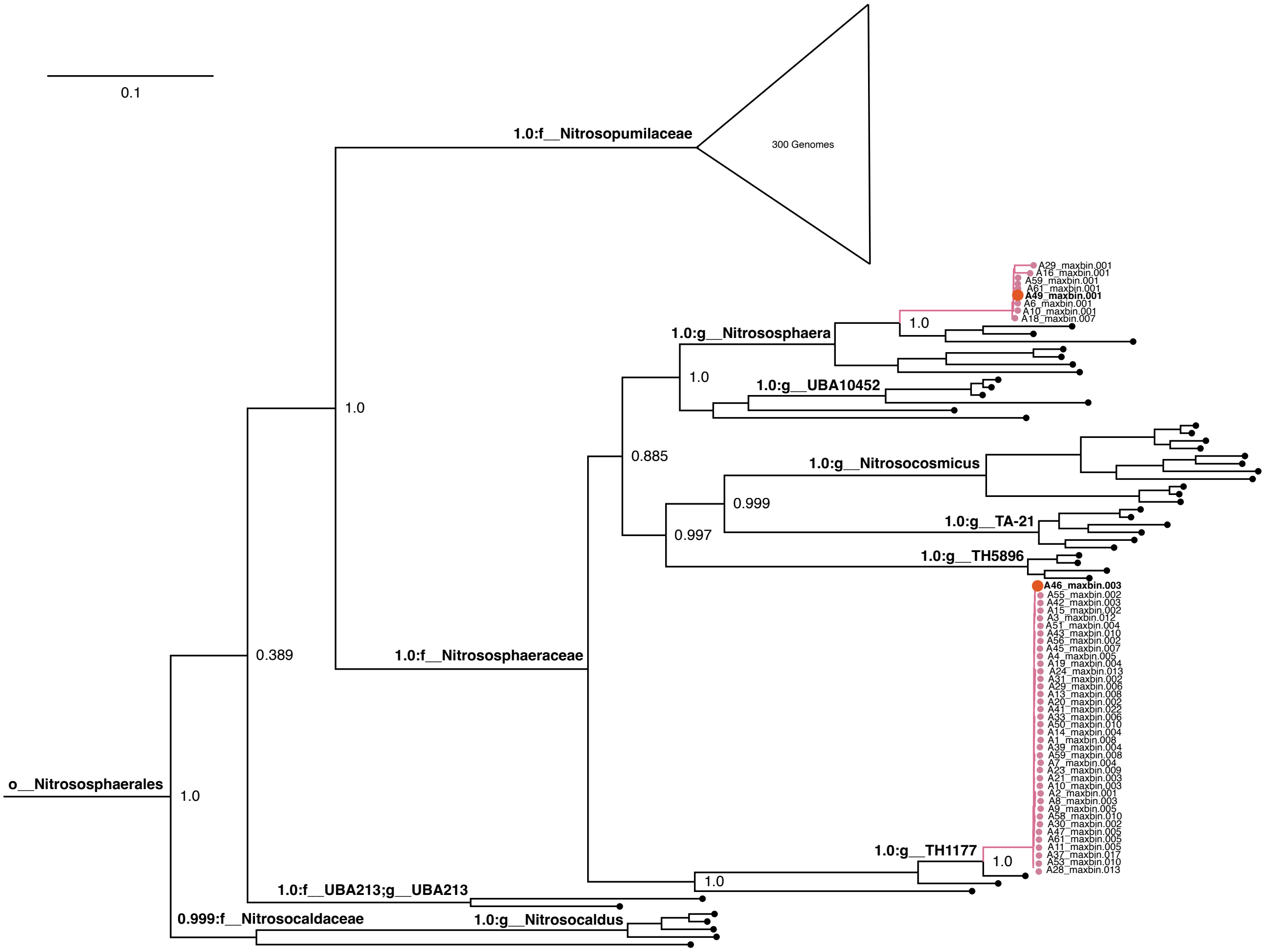

Supplementary Figure SF8. Relative abundances of the MAGs representative for (a) *Nitrososphaeraceae* TH1177 (A46_maxbin.003) and (b) *Nitrososphaera* sp. (A49_maxbin.001) in aggregates; (c) quantification of ammonia-oxidizing archaea (AOA) in different aggregate fractions; and Spearman's correlations of AOA copy numbersfor (d) *Nitrososphaeraceae* TH1177 (A46_maxbin.003) and (b) *Nitrososphaera* sp. (A49_maxbin.001) in aggregates of different sizes. Different letters in the boxplots represent significant differences ($P < 0.05$) between aggregate size fractions.

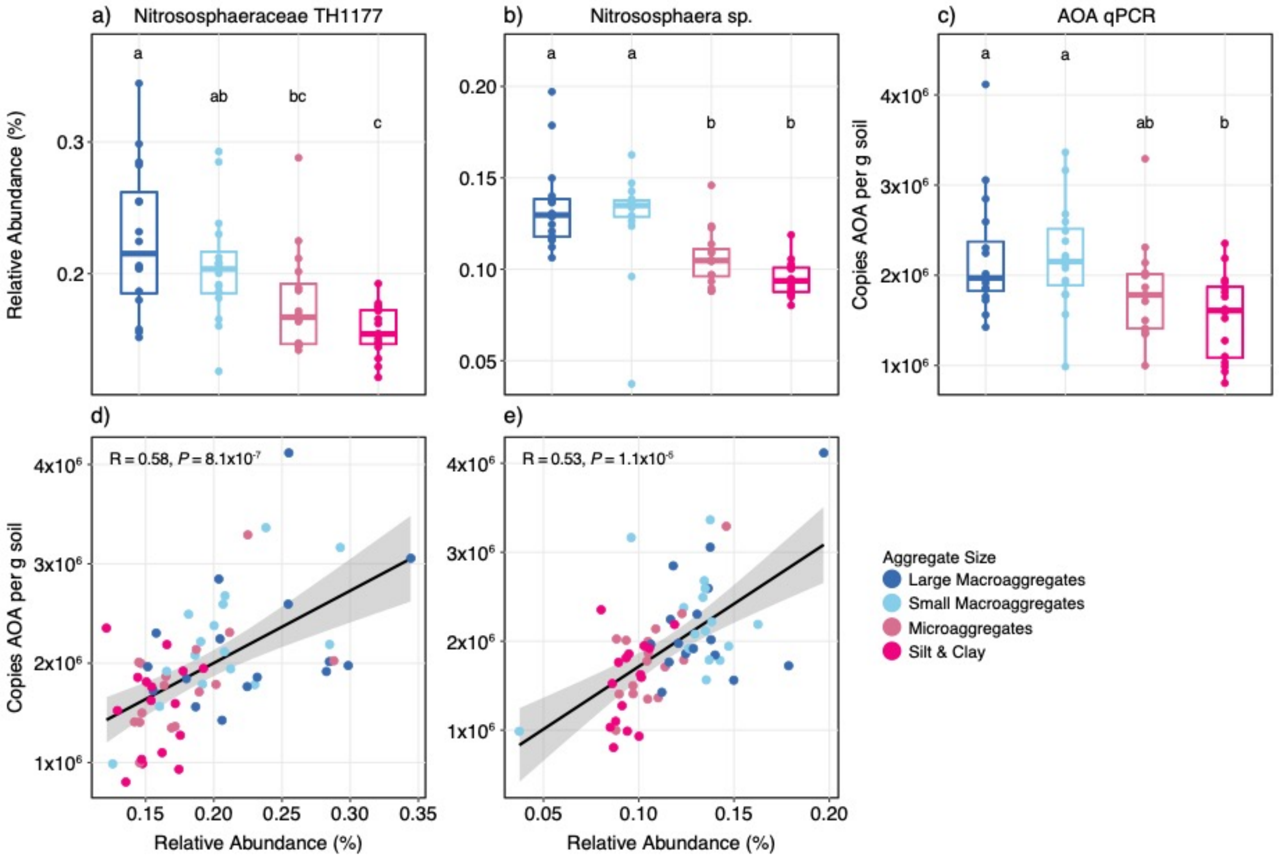

Supplement: Supplementary_material_ycag161 [file supplementary_material_ycag161.zip › ISMECOMMUN-D-25-00448R1_Supplementary_CleanCopy.pdf]
