# Supplementary material for: Multi-omics resolved integration reveals microbial niche separation in soil aggregates
Source: ISME Commun. 2026 Jun 11;6(1):ycag161. doi: 10.1093/ismeco/ycag161 (PMC13398701; doi:10.1093/ismeco/ycag161)
Supplement: Supplementary_material_ycag161 [file supplementary_material_ycag161.zip › SupplementaryTable_ST4.docx]

**Supplementary Table ST3**. Differentially abundant KEGG genes (*P* < 0.05) between aggregate size fractions. A positive log_2_-fold change value indicates enrichment in the microaggregates and silt & clay, while a negative log_2_-fold change value indicates enrichment in the large and small macroaggregates.

| **KEGG ID** | **Log_2_FC** | **Log_2_CPM** | **LR** | ***P*-value** | **FDR** | **Gene Name** |
| --- | --- | --- | --- | --- | --- | --- |
| K00127 | -0.49 | 6.04 | 24.57 | 7.15E-07 | 8.11E-06 | formate subunit gamma |
| K00150 | -0.41 | 6.30 | 22.44 | 2.17E-06 | 2.01E-05 | glyceraldehyde-3-dehydrogenase (NAD(P)) [EC:1.2.1.59] |
| K00232 | 0.46 | 7.65 | 35.00 | 3.29E-09 | 9.66E-08 | acyl-oxidase [EC:1.3.3.6] |
| K00370 | 0.61 | 6.42 | 32.57 | 1.15E-08 | 2.68E-07 | nitrate reductase / oxidoreductase, subunit [EC:1.7.5.1.7.99.-] |
| K00371 | 0.51 | 6.73 | 55.67 | 8.57E-14 | 1.31E-11 | nitrate reductase / oxidoreductase, subunit [EC:1.7.5.1.7.99.-] |
| K00450 | -0.53 | 6.75 | 46.97 | 7.20E-12 | 6.64E-10 | gentisate 1,2-dioxygenase [EC:1.13.11.4] |
| K00496 | 0.67 | 6.16 | 52.99 | 3.35E-13 | 4.30E-11 | alkane 1-monooxygenase [EC:1.14.15.3] |
| K00505 | -0.57 | 6.32 | 46.93 | 7.35E-12 | 6.64E-10 | tyrosinase [EC:1.14.18.1] |
| K00728 | 0.44 | 8.51 | 44.87 | 2.10E-11 | 1.77E-09 | dolichyl-phosphate-mannose-mannosyltransferase [EC:2.4.1.109] |
| K00758 | 0.44 | 6.23 | 24.97 | 5.81E-07 | 6.79E-06 | thymidine phosphorylase [EC:2.4.2.4] |
| K00868 | 0.52 | 6.23 | 24.97 | 5.82E-07 | 6.79E-06 | pyridoxine kinase [EC:2.7.1.35] |
| K01153 | -0.47 | 6.43 | 42.79 | 6.09E-11 | 4.01E-09 | type restriction enzyme, subunit [EC:3.1.21.3] |
| K01301 | -0.46 | 5.93 | 6.68 | 9.77E-03 | 2.46E-02 | N-acetylated-alpha-acidic dipeptidase [EC:3.4.17.21] |
| K01302 | -0.41 | 7.83 | 11.90 | 5.62E-04 | 2.27E-03 | carboxypeptidase Q [EC:3.4.17.-] |
| K01428 | -0.44 | 6.84 | 25.01 | 5.70E-07 | 6.78E-06 | urease alpha [EC:3.5.1.5] |
| K01467 | -0.51 | 5.72 | 7.74 | 5.41E-03 | 1.52E-02 | beta-class C [EC:3.5.2.6] |
| K01576 | -0.46 | 6.15 | 43.80 | 3.63E-11 | 2.68E-09 | benzoylformate decarboxylase [EC:4.1.1.7] |
| K01886 | -0.55 | 6.48 | 21.55 | 3.45E-06 | 2.96E-05 | glutaminyl-synthetase [EC:6.1.1.18] |
| K01958 | 0.42 | 6.65 | 13.10 | 2.95E-04 | 1.31E-03 | pyruvate carboxylase [EC:6.4.1.1] |
| K01989 | -0.48 | 8.58 | 60.63 | 6.87E-15 | 2.10E-12 | putative tryptophan/transport substrate-protein |
| K02005 | -0.40 | 8.42 | 14.00 | 1.83E-04 | 8.86E-04 | HlyD secretion protein |
| K02014 | -0.50 | 9.96 | 7.16 | 7.47E-03 | 1.97E-02 | iron outermembrane protein |
| K02117 | -0.44 | 6.94 | 18.16 | 2.03E-05 | 1.38E-04 | V/A-H+/Na+-ATPase A [EC:7.1.2.7.2.2.1] |
| K02172 | -0.50 | 8.10 | 16.15 | 5.86E-05 | 3.39E-04 | bla protein blaR1 |
| K02322 | -0.52 | 7.86 | 27.73 | 1.40E-07 | 2.17E-06 | DNA II subunit [EC:2.7.7.7] |
| K02448 | -0.45 | 6.21 | 15.52 | 8.15E-05 | 4.49E-04 | nitric reductase protein |
| K02453 | -0.67 | 5.33 | 16.70 | 4.38E-05 | 2.64E-04 | general pathway D |
| K02481 | -0.55 | 7.52 | 24.04 | 9.44E-07 | 1.01E-05 | two-system, family, regulator |
| K02517 | -0.54 | 6.01 | 14.25 | 1.60E-04 | 7.94E-04 | Kdo2-IVA lauroyltransferase/acyltransferase [EC:2.3.1.2.3.1.-] |
| K02558 | -0.52 | 5.80 | 11.63 | 6.50E-04 | 2.58E-03 | UDP-N-acetylmuramate: L-alanyl-gamma-D-glutamyl-meso-ligase [EC:6.3.2.45] |
| K02668 | -0.41 | 6.18 | 12.07 | 5.13E-04 | 2.12E-03 | two-system, family, histidine PilS [EC:2.7.13.3] |
| K02674 | -0.50 | 6.30 | 15.72 | 7.35E-05 | 4.10E-04 | type pilus protein PilY1 |
| K02683 | -0.43 | 6.31 | 24.40 | 7.84E-07 | 8.69E-06 | DNA small subunit [EC:2.7.7.102] |
| K02800 | 0.56 | 7.17 | 56.03 | 7.13E-14 | 1.16E-11 | mannitol system or component [EC:2.7.1.197] |
| K03042 | -0.45 | 7.99 | 27.93 | 1.26E-07 | 1.96E-06 | DNA-RNA subunit A" [EC:2.7.7.6] |
| K03296 | -0.59 | 7.24 | 9.57 | 1.98E-03 | 6.61E-03 | hydrophobic/exporter-1 (G- bacteria), family |
| K03299 | 0.44 | 7.79 | 35.86 | 2.12E-09 | 6.55E-08 | gluconate:H+ symporter, family |
| K03367 | -1.27 | 5.34 | 21.09 | 4.38E-06 | 3.68E-05 | D-alanine--poly(phosphoribitol) subunit 1 [EC:6.1.1.13] |
| K03427 | -0.54 | 6.59 | 78.11 | 9.73E-19 | 7.91E-16 | type restriction M protein [EC:2.1.1.72] |
| K03566 | -0.42 | 6.82 | 5.59 | 1.80E-02 | 4.03E-02 | LysR transcriptional regulator, cleavage transcriptional activator |
| K03583 | 0.40 | 8.08 | 19.49 | 1.01E-05 | 7.51E-05 | exodeoxyribonuclease gamma subunit [EC:3.1.11.5] |
| K03648 | 0.45 | 6.49 | 28.04 | 1.19E-07 | 1.90E-06 | uracil-glycosylase [EC:3.2.2.27] |
| K03654 | -0.66 | 6.09 | 11.04 | 8.90E-04 | 3.39E-03 | ATP-DNA RecQ [EC:3.6.4.12] |
| K03694 | -0.57 | 5.48 | 8.76 | 3.08E-03 | 9.56E-03 | ATP-Clp ATP-subunit ClpA |
| K04034 | -0.54 | 7.40 | 25.10 | 5.45E-07 | 6.58E-06 | anaerobic magnesium-IX ester cyclase [EC:1.21.98.3] |
| K04070 | -0.43 | 6.42 | 42.25 | 8.03E-11 | 4.90E-09 | putative formate activating enzyme [EC:1.97.1.4] |
| K04102 | -0.54 | 7.69 | 81.89 | 1.44E-19 | 1.75E-16 | 4,5-decarboxylase [EC:4.1.1.55] |
| K05341 | 0.50 | 7.08 | 25.67 | 4.05E-07 | 5.22E-06 | amylosucrase [EC:2.4.1.4] |
| K05365 | -0.67 | 5.43 | 13.41 | 2.50E-04 | 1.14E-03 | penicillin-protein 1B [EC:2.4.1.3.4.16.4] |
| K05830 | -0.41 | 6.30 | 13.52 | 2.37E-04 | 1.09E-03 | LysW-gamma-L-lysine/LysW-L-aminotransferase [EC:2.6.1.2.6.1.-] |
| K05831 | -0.52 | 5.98 | 10.52 | 1.18E-03 | 4.31E-03 | DEFINITION [group protein]-lysine/hydrolase [EC:3.5.1.3.5.1.132] |
| K05970 | -0.52 | 5.65 | 15.82 | 6.98E-05 | 3.94E-04 | sialate O-acetylesterase [EC:3.1.1.53] |
| K06006 | -0.45 | 6.76 | 17.35 | 3.12E-05 | 2.02E-04 | periplasmic CpxP/Spy |
| K06151 | -0.49 | 6.33 | 24.26 | 8.43E-07 | 9.17E-06 | gluconate 2-alpha chain [EC:1.1.99.3] |
| K06160 | -1.14 | 4.55 | 18.85 | 1.42E-05 | 1.00E-04 | putative transport ATP-binding/protein |
| K06219 | 0.47 | 6.27 | 21.01 | 4.57E-06 | 3.78E-05 | S-adenosylmethionine-methyltransferase |
| K06894 | -1.05 | 7.13 | 19.71 | 9.01E-06 | 6.78E-05 | alpha-2-macroglobulin |
| K06895 | 0.50 | 5.99 | 17.15 | 3.45E-05 | 2.19E-04 | L-exporter protein LysE/ArgO |
| K06909 | -0.73 | 6.26 | 64.58 | 9.27E-16 | 3.77E-13 | phage large subunit |
| K06965 | -0.40 | 6.30 | 15.50 | 8.24E-05 | 4.53E-04 | protein pelota |
| K06989 | -0.41 | 6.72 | 57.73 | 3.00E-14 | 6.65E-12 | aspartate dehydrogenase [EC:1.4.1.21] |
| K07003 | -0.47 | 6.83 | 12.41 | 4.26E-04 | 1.80E-03 | uncharacterized protein |
| K07165 | -0.66 | 7.19 | 13.74 | 2.09E-04 | 9.90E-04 | transmembrane sensor |
| K07277 | -0.41 | 8.01 | 13.08 | 2.98E-04 | 1.32E-03 | outer protein porin family |
| K07303 | -0.62 | 6.57 | 11.24 | 8.03E-04 | 3.12E-03 | isoquinoline 1-subunit beta [EC:1.3.99.16] |
| K07689 | -0.42 | 5.82 | 14.05 | 1.78E-04 | 8.67E-04 | two-system, family, response UvrY |
| K07705 | -0.54 | 6.36 | 19.13 | 1.22E-05 | 8.87E-05 | two-system, family, regulator LytT |
| K07713 | -0.51 | 7.65 | 21.02 | 4.53E-06 | 3.77E-05 | two-system, family, regulator HydG |
| K07714 | -0.51 | 8.63 | 20.92 | 4.80E-06 | 3.90E-05 | two-system, family, regulator AtoC |
| K07768 | 0.48 | 6.55 | 22.48 | 2.12E-06 | 1.97E-05 | two-system, family, histidine SenX3 [EC:2.7.13.3] |
| K07787 | -1.08 | 5.58 | 16.56 | 4.72E-05 | 2.81E-04 | copper/efflux protein |
| K07795 | -0.44 | 10.50 | 24.72 | 6.63E-07 | 7.62E-06 | putative transport protein |
| K08191 | -0.70 | 5.49 | 22.35 | 2.27E-06 | 2.09E-05 | MFS transporter, family, transporter |
| K08303 | -0.64 | 5.56 | 15.90 | 6.67E-05 | 3.78E-04 | U32 peptidase [EC:3.4.-.-] |
| K08372 | 0.47 | 7.50 | 18.97 | 1.33E-05 | 9.60E-05 | putative protease PepD [EC:3.4.21.-] |
| K08676 | -0.78 | 7.15 | 10.93 | 9.44E-04 | 3.56E-03 | tricorn protease [EC:3.4.21.-] |
| K09136 | -0.41 | 7.39 | 46.13 | 1.11E-11 | 9.63E-10 | ribosomal S12 accessory factor |
| K09252 | -0.80 | 5.11 | 15.11 | 1.01E-04 | 5.39E-04 | feruloyl esterase [EC:3.1.1.73] |
| K09781 | 0.45 | 6.71 | 35.89 | 2.09E-09 | 6.55E-08 | uncharacterized protein |
| K09809 | 0.42 | 7.28 | 12.82 | 3.43E-04 | 1.48E-03 | CDP-glycerophosphotransferase [EC:2.7.8.12] |
| K09861 | 0.53 | 6.53 | 33.26 | 8.08E-09 | 2.05E-07 | uncharacterized protein |
| K10007 | 0.47 | 6.32 | 22.52 | 2.08E-06 | 1.95E-05 | glutamate system protein |
| K10532 | -0.54 | 6.15 | 21.57 | 3.42E-06 | 2.94E-05 | heparan-alpha-N-acetyltransferase [EC:2.3.1.78] |
| K10843 | 0.49 | 7.31 | 36.98 | 1.20E-09 | 4.23E-08 | DNA repair ERCC-3 [EC:3.6.4.12] |
| K11021 | -1.32 | 5.80 | 34.53 | 4.20E-09 | 1.18E-07 | insecticidal complex TccC |
| K11085 | -0.77 | 5.76 | 17.74 | 2.53E-05 | 1.67E-04 | ATP-cassette, B, MsbA [EC:7.5.2.6] |
| K11089 | -0.51 | 5.90 | 22.03 | 2.68E-06 | 2.42E-05 | 60 SS-A/ribonucleoprotein |
| K11414 | 0.52 | 6.20 | 29.96 | 4.42E-08 | 7.98E-07 | NAD+-protein sirtuin 4 [EC:2.3.1.286] |
| K11891 | -0.62 | 6.29 | 14.33 | 1.53E-04 | 7.71E-04 | type secretion protein ImpL |
| K11893 | -0.48 | 5.57 | 12.93 | 3.23E-04 | 1.41E-03 | type secretion protein ImpJ |
| K11895 | -0.45 | 5.31 | 11.61 | 6.54E-04 | 2.59E-03 | type secretion protein ImpH |
| K11904 | -0.61 | 7.40 | 14.71 | 1.26E-04 | 6.50E-04 | type secretion secreted VgrG |
| K12444 | -1.43 | 6.80 | 29.76 | 4.88E-08 | 8.68E-07 | phthiocerol/synthesis type-polyketide E [EC:2.3.1.292] |
| K12503 | 0.41 | 6.34 | 22.57 | 2.03E-06 | 1.91E-05 | short-Z-diphosphate synthase [EC:2.5.1.68] |
| K13017 | -0.43 | 6.44 | 18.13 | 2.06E-05 | 1.40E-04 | UDP-2-acetamido-2-deoxy-ribo-aminotransferase [EC:2.6.1.98] |
| K13288 | 0.41 | 6.90 | 38.17 | 6.49E-10 | 2.68E-08 | oligoribonuclease [EC:3.1.-.-] |
| K13572 | 0.44 | 6.79 | 25.54 | 4.34E-07 | 5.52E-06 | proteasome factor B |
| K13573 | 0.40 | 6.55 | 22.74 | 1.86E-06 | 1.79E-05 | proteasome factor C |
| K13992 | -0.64 | 6.27 | 19.40 | 1.06E-05 | 7.77E-05 | photosynthetic center c subunit |
| K14055 | -0.50 | 6.21 | 13.61 | 2.25E-04 | 1.04E-03 | universal protein E |
| K14645 | -0.48 | 8.12 | 41.70 | 1.07E-10 | 6.04E-09 | serine protease [EC:3.4.21.-] |
| K14954 | 0.45 | 6.28 | 42.18 | 8.33E-11 | 4.95E-09 | lipoprotein LprG |
| K14986 | -0.47 | 7.64 | 17.93 | 2.29E-05 | 1.55E-04 | two-system, family, kinase FixL [EC:2.7.13.3] |
| K15016 | -0.44 | 6.52 | 14.80 | 1.19E-04 | 6.23E-04 | enoyl-hydratase / 3-hydroxyacyl-dehydrogenase [EC:4.2.1.1.1.1.35] |
| K15019 | -0.51 | 6.19 | 36.22 | 1.76E-09 | 5.71E-08 | 3-hydroxypropionyl-A dehydratase [EC:4.2.1.116] |
| K15532 | -0.46 | 6.08 | 12.08 | 5.09E-04 | 2.11E-03 | unsaturated hydrolase [EC:3.2.1.172] |
| K15836 | -0.56 | 7.09 | 13.15 | 2.88E-04 | 1.28E-03 | formate transcriptional activator |
| K16090 | -0.56 | 5.86 | 9.46 | 2.10E-03 | 6.91E-03 | catecholate receptor |
| K16163 | 0.50 | 6.41 | 35.87 | 2.11E-09 | 6.55E-08 | maleylpyruvate isomerase [EC:5.2.1.4] |
| K16554 | -0.65 | 6.29 | 11.36 | 7.50E-04 | 2.93E-03 | polysaccharide transport protein |
| K16649 | 0.59 | 6.53 | 34.28 | 4.78E-09 | 1.29E-07 | rhamnopyranosyl-N-acetylglucosaminyl-diphospho-beta-1,3/1,4-galactofuranosyltransferase [EC:2.4.1.287] |
| K16650 | 0.63 | 6.78 | 39.80 | 2.81E-10 | 1.37E-08 | galactofuranosylgalactofuranosylrhamnosyl-N-acetylglucosaminyl-diphospho-beta-1,5/1,6-galactofuranosyltransferase [EC:2.4.1.288] |
| K16842 | -0.40 | 6.70 | 38.03 | 6.96E-10 | 2.75E-08 | allantoinase [EC:3.5.2.5] |
| K16874 | -0.46 | 7.11 | 66.69 | 3.18E-16 | 1.55E-13 | 2,5-decarboxylase 1 |
| K17713 | -0.55 | 8.96 | 16.88 | 3.99E-05 | 2.48E-04 | outer protein factor BamB |
| K17734 | -0.49 | 7.13 | 10.80 | 1.01E-03 | 3.79E-03 | serine AprX [EC:3.4.21.-] |
| K17758 | -0.64 | 5.30 | 13.52 | 2.36E-04 | 1.09E-03 | ADP-NAD(P)H-dehydratase [EC:4.2.1.136] |
| K17837 | -0.55 | 6.11 | 14.39 | 1.49E-04 | 7.51E-04 | metallo-beta-class B [EC:3.5.2.6] |
| K18068 | -0.79 | 6.23 | 36.32 | 1.68E-09 | 5.52E-08 | phthalate 4,5-dioxygenase [EC:1.14.12.7] |
| K18138 | -0.52 | 6.40 | 6.44 | 1.11E-02 | 2.75E-02 | multidrug pump |
| K18139 | -0.56 | 6.73 | 9.15 | 2.48E-03 | 7.89E-03 | outer protein, efflux system |
| K18455 | 0.40 | 6.71 | 24.81 | 6.34E-07 | 7.36E-06 | mycothiol S-amidase [EC:3.5.1.115] |
| K18479 | 0.49 | 6.13 | 16.16 | 5.82E-05 | 3.38E-04 | sulfoquinovose isomerase [EC:5.3.1.31] |
| K18481 | 0.59 | 7.22 | 35.73 | 2.26E-09 | 6.81E-08 | Mce-membrane protein |
| K18578 | -0.48 | 5.90 | 18.87 | 1.40E-05 | 9.95E-05 | xyloglucan-exo-beta-1,4-glucanase [EC:3.2.1.155] |
| K18601 | -0.51 | 6.78 | 21.66 | 3.26E-06 | 2.83E-05 | aldehyde dehydrogenase [EC:1.2.1.-] |
| K18691 | -0.42 | 5.63 | 8.87 | 2.90E-03 | 9.08E-03 | membrane-lytic transglycosylase F [EC:4.2.2.-] |
| K18926 | 0.49 | 6.27 | 8.41 | 3.73E-03 | 1.12E-02 | MFS transporter, family, resistance protein |
| K19092 | -0.61 | 5.83 | 32.62 | 1.12E-08 | 2.68E-07 | toxin ParE1/3/4 |
| K19701 | -0.47 | 6.90 | 12.06 | 5.14E-04 | 2.12E-03 | aminopeptidase YwaD [EC:3.4.11.3.4.11.10] |
| K21084 | -0.51 | 6.81 | 12.38 | 4.33E-04 | 1.83E-03 | diguanylate cyclase [EC:2.7.7.65] |
| K21572 | -0.81 | 8.13 | 20.34 | 6.49E-06 | 5.05E-05 | starch-outer protein, SusD/family |
| K21573 | -2.90 | 6.29 | 29.19 | 6.55E-08 | 1.12E-06 | TonB-starch-outer protein SusC |
| K21688 | 0.54 | 6.74 | 32.85 | 9.98E-09 | 2.43E-07 | resuscitation-factor RpfB |
| K22447 | -0.42 | 7.84 | 36.79 | 1.32E-09 | 4.53E-08 | archaeal chaperonin |
| K22476 | 0.43 | 6.70 | 43.43 | 4.39E-11 | 3.15E-09 | N-synthase [EC:2.3.1.1] |
| K22486 | -0.71 | 5.94 | 11.03 | 8.98E-04 | 3.42E-03 | transcriptional HilA, transcriptional of SPI1 |
| K22708 | 0.40 | 6.69 | 13.73 | 2.11E-04 | 9.93E-04 | poly(ribitol-phosphate) beta-N-acetylglucosaminyltransferase [EC:2.4.1.355] |
| K22769 | 0.51 | 6.56 | 21.91 | 2.86E-06 | 2.55E-05 | NADPH-stearoyl-9-desaturase [EC:1.14.19.-] |
| K22770 | 0.47 | 7.09 | 26.20 | 3.08E-07 | 4.26E-06 | stearoyl-9-NADPH oxidoreductase |
| K23424 | -0.71 | 6.06 | 22.03 | 2.69E-06 | 2.42E-05 | protein O-mannosyl-transferase [EC:2.4.1.-] |
| K23842 | 0.47 | 6.52 | 31.77 | 1.73E-08 | 3.72E-07 | NAD(P)dehydrogenase (quinone) [EC:1.6.5.2] |
| K23980 | 0.44 | 6.82 | 32.08 | 1.48E-08 | 3.28E-07 | cysteinylglycine-S-dipeptidase [EC:3.4.13.23] |
| K24017 | 0.54 | 6.10 | 27.55 | 1.53E-07 | 2.33E-06 | phosphoribosyl A [EC:5.3.1.5.3.1.24] |
